# Supplementary material for: Transneuronal delivery of hyper-interleukin-6 enables functional recovery after severe spinal cord injury in mice
Source: Nat Commun. 2021 Jan 15;12:391. doi: 10.1038/s41467-020-20112-4 (PMC7810685; doi:10.1038/s41467-020-20112-4)
Supplement: Supplementary file 1 — Supplementary Information [file 41467_2020_20112_MOESM1_ESM.pdf]

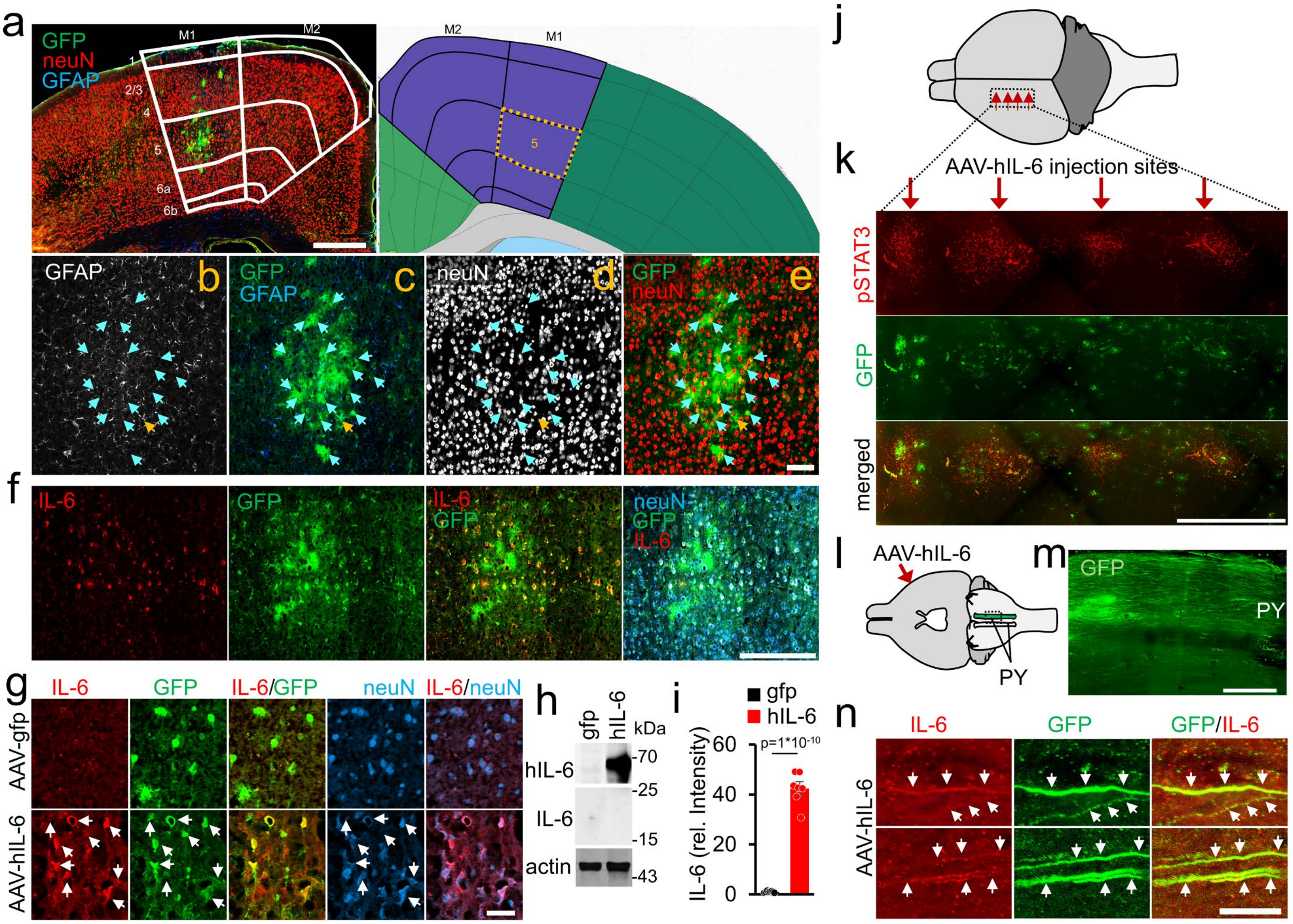

Supplementary Figure 1: Validation of cortical AAV2 transduction

a) Coronal section showing the left hemisphere of the sensorimotor cortex 3 weeks after AAV2-hIL-6 injection stained for glial fibrillary acid protein (GFAP, blue), NeuN (red), and GFP (green). White boxes indicate layers of primary (M1) and secondary (M2) motor cortex in an overlay with a corresponding map from *Allen Brain Atlas,* as shown for the right hemisphere. Scale bar: 500 µm.

b-e) Higher magnification of the image shown in A. Blue arrows mark neuN positive neurons, while orange arrows indicate GFAP positive astrocytes. Scale bar: 50 µm.

f) Coronal section of the sensorimotor cortex, as described in A, stained for hIL-6 (IL-6, red), GFP (green), and NeuN (blue). Scale bar: 200 µm.

g) Cortical sections showing AAV2-GFP or AAV2-hIL-6 transduced (GFP, green) layer 5 cortical motor neurons immunohistochemically stained for neuN (blue) and hIL-6 (IL-6, red). In contrast to AAV-GFP transduction, AAV-hIL-6 treatment resulted in hIL-6 protein expression. Scale bar: 50 µm.

h) Western blot analysis of cortical lysates from AAV2-gfp (gfp), or AAV2-hIL-6 (hIL-6) treated mice. IL-6 antibody staining shows, only for the AAV2-hIL6 treated group, a clear band at ~70 kDa, the size of the hIL-6 as a fusion protein with the IL-6 receptor. No band natural IL-6 protein at ~20 kDa was observed. Beta-actin served as a loading control.

i) Quantification of the hIL-6 band in western blots shown in H. Pooled samples from 8 (GFP) and 7 (hIL-6) animals.

j) Schematic drawing of a mouse brain with cortical AAV-injection sites (red arrows). The dotted box shows the location of the immunohistochemical staining shown in **k.**

k) Optical section through 100 µm of cleared motor cortex layer V from a mouse 4 days after AAV-hIL-6 injections. Staining for phosphorylated STAT3 (pSTAT3, red) and GFP indicates activation of the JAK/STAT3 pathway at this early time point after intracortical injection. Scale bar: 500 µm.

l) Schematic illustration related to images shown in **m** and **n:** Bottom view of the mouse brain showing the pyramidal tracts (PY) in the medulla. Axons of GFP-expressing transduced cortical neurons in the left pyramid are indicated in green. The dotted box illustrates the area used for confocal scans from cleared tissue, as shown in **m**. The red arrow indicates a unilateral (left) intracortical AAV-hIL-6 injection.

m) Maximum intensity projection of transverse confocal tile scan through cleared mouse brain tissue with GFP (green) positive axons. Scans were performed 3 weeks after unilateral (left) intracortical injection of AAV2-hIL-6**,** using 250 x magnification on a Leica SP8 confocal microscope. Scale bar: 250 µm.

n) Maximum intensity projection of high-resolution scans through cleared mouse brain tissue as depicted in **l**, using a 630 x magnification on a Leica SP8 confocal microscope. Tissue was stained for GFP (green) and hIL-6 (IL-6, red). hIL-6 has only been detected in GFP positive axons as indicated by white arrows. Scale bar: 25 µm.

Significances of intergroup differences in **i** were evaluated using the student’s t-test and are indicated by p-value.

Source data are provided as a Source Data file.

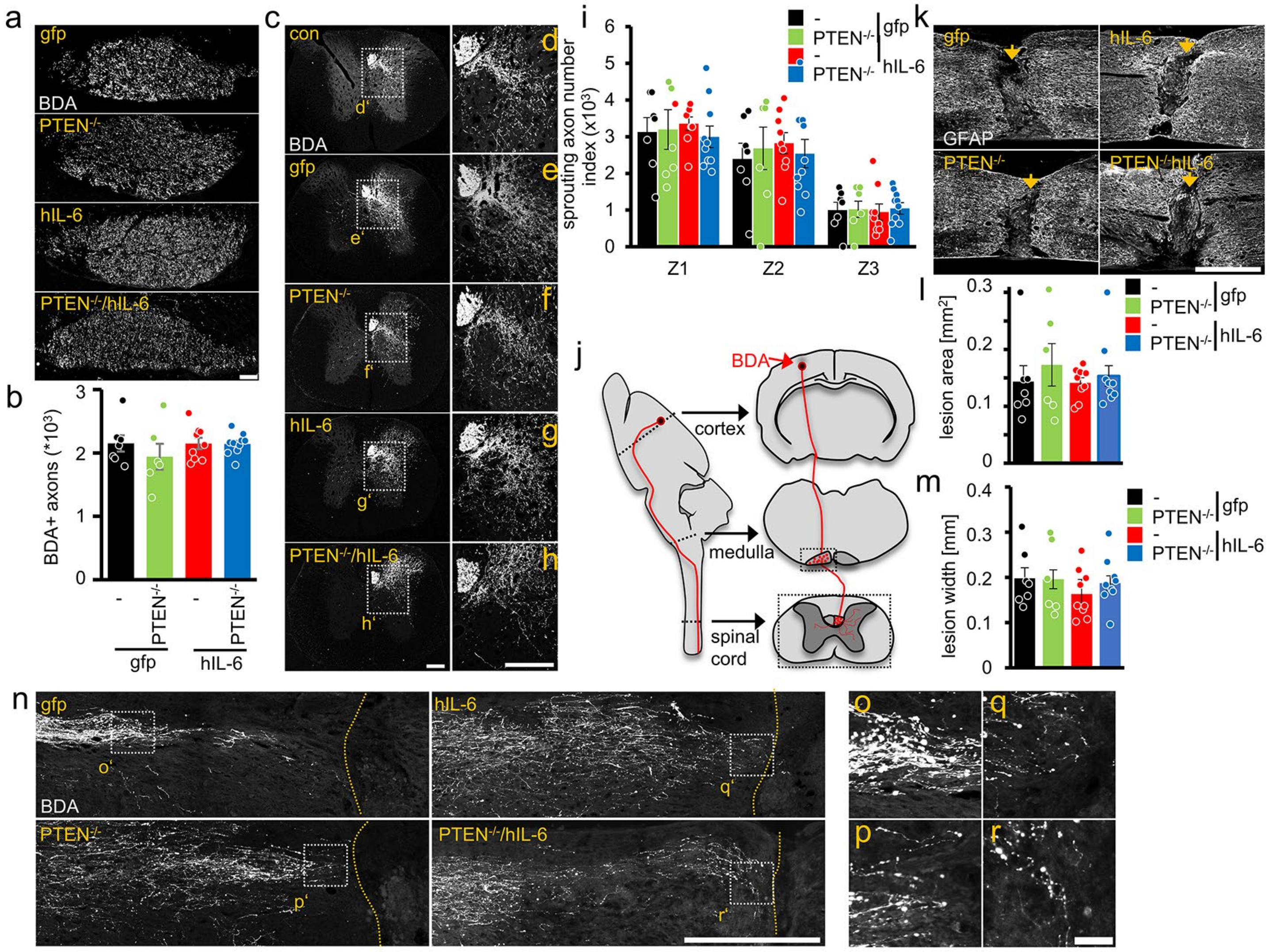

Supplementary Figure 2: hIL-6 does not affect proximal CST axon sprouting

a) Coronal sections of the medullary pyramid with BDA-labeled CST axons in $PTEN^{f/f}$ mice 8 weeks after severe spinal cord crush (SCC) as described in **Fig. 2 a**. Scale bar: 50 µm.

b) Quantification of the density of BDA-labeled CST-axons in sections as described in **a**. Counts of five sections were averaged per animal. Values represent means +/- SEM of 5-9 animals per group ($PTEN^{+/+}$/gfp, n=7; $PTEN^{+/+}$/hIL-6, n=9; $PTEN^{-/-}$/gfp, n=6; $PTEN^{-/-}$/hIL-6, n=10).

c) Representative images with BDA-labeled CST-axons in transverse spinal cord sections 3 mm rostral to the lesion site in animals as described in **a** and additional wild-type mice without SCC (con). Scale bar: 100 µm.

d-h) Higher magnification of dashed boxes from images presented in **c**. Scale bar: 200 µm.

i) Quantification of axons sprouting into the ipsilateral gray matter at defined distances from the midline (Z1-3). Counts of 5 sections were averaged per animal. Values represent means +/- SEM of 5-9 animals per group ($PTEN^{+/+}$/gfp, n=7; $PTEN^{+/+}$/hIL-6, n=9; $PTEN^{-/-}$/gfp, n=6; $PTEN^{-/-}$/hIL-6, n=10).

j) Schematic drawing illustrating the location of coronal sections of the medulla and spinal cord shown in **a** and **c** (dotted boxes).

k) Representative images of sagittal spinal cord sections from mice, as described in **a.** Lesion sites (arrows), were visualized by the absence of GFAP positive astrocytes. Scale bar: 500 µm.

l/m) Quantification of the lesion area (**l**) and average width (**m**) in sections as described in **k**. Values represent means +/- SEM of 65-9 animals per group ($PTEN^{+/+}$/gfp, n=7; $PTEN^{+/+}$/hIL-6, n=9; $PTEN^{-/-}$/gfp, n=6; $PTEN^{-/-}$/hIL-6, n=10).

n) Images of BDA labeled CST axons rostral to the lesion site (dotted line) in sagittal spinal cord sections of mice treated as described in Fig. 2 A. Scale bar: 500 µm.

o-r) Higher magnifications of areas as indicated in **n** showing pronounced retraction bulbs in the AAV-gfp treated control group. Scale bar: 50 µm.

Statistics in **b, i, l,** and **m** were evaluated using two-way analysis of variance (ANOVA) with Holm-Sidak and Tukey post hoc tests, showing no significant differences between the groups.

Source data are provided as a Source Data file.

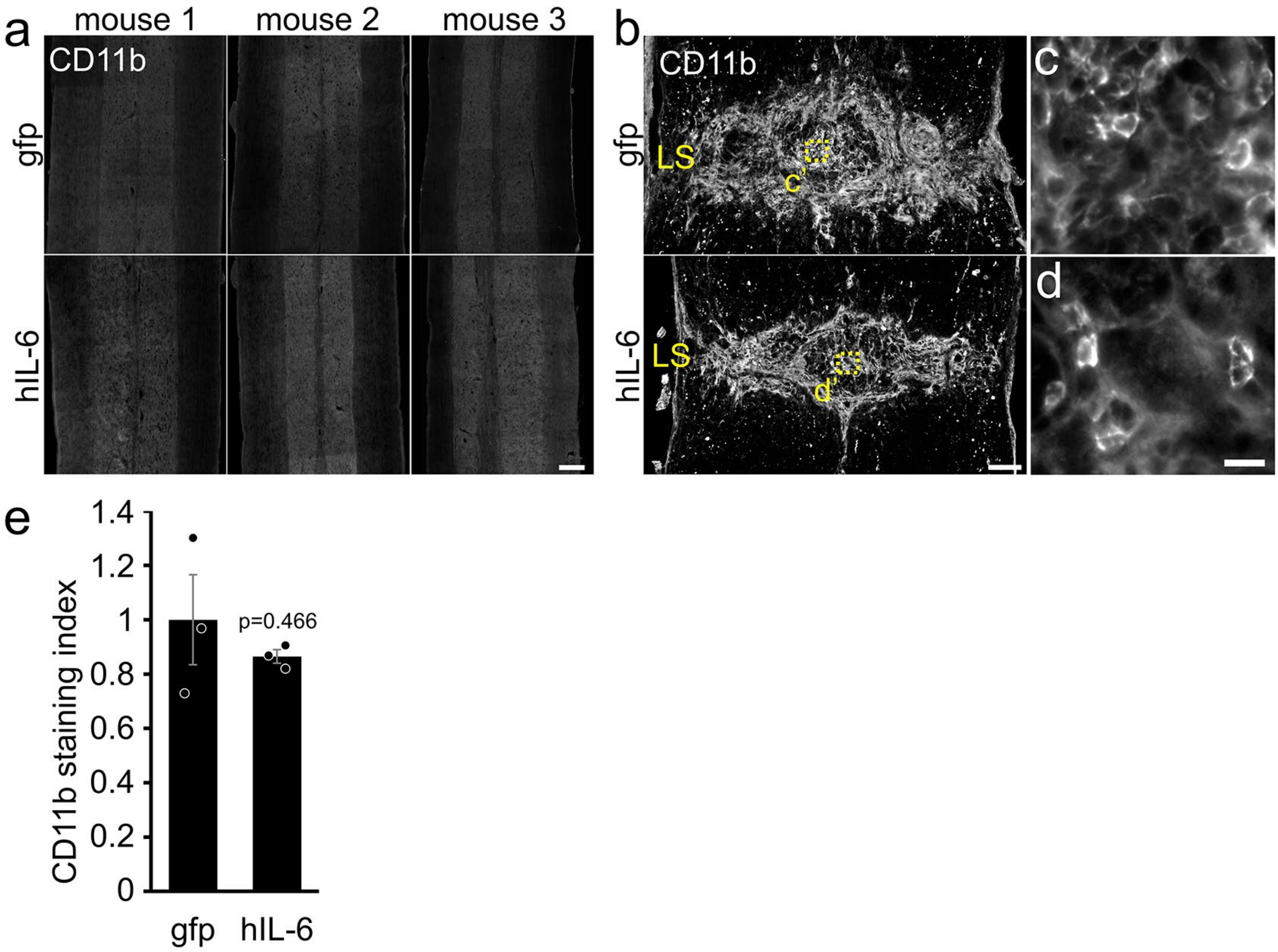

Supplementary Figure 3: Cortical hIL-6 expression does not activate spinal macrophages/microglia

a) Immunohistochemical CD11b staining in sagittal sections of the uninjured thoracic spinal cord from 3 mice treated with either AAV2-gfp or 3 mice treated with AAV2-hIL-6. Scale bar: 50 µm.

b) Images of lesion sites (LS) in coronal spinal cord sections 8 weeks after T8 severe spinal cord crush and intracortical AAV2-hIL-6 (hIL-6) or AAV2-gfp (gfp) application as described in **Fig. 2 a**. Activated macrophages and microglia cells are immunohistochemically stained for CD11b. Scale bar: 50 µm.

c-d) Higher magnification of areas as indicated in B. Scale bar: 10 µm.

e) Quantification of CD11b staining in spinal cord lesion sites of AAV-hIL-6 (n=3) or AAV-gfp (n=3) treated animals as depicted in **c-d**. Significances of intergroup differences were evaluated using the student's t-test and are indicated by p-value.

Source data are provided as a Source Data file.

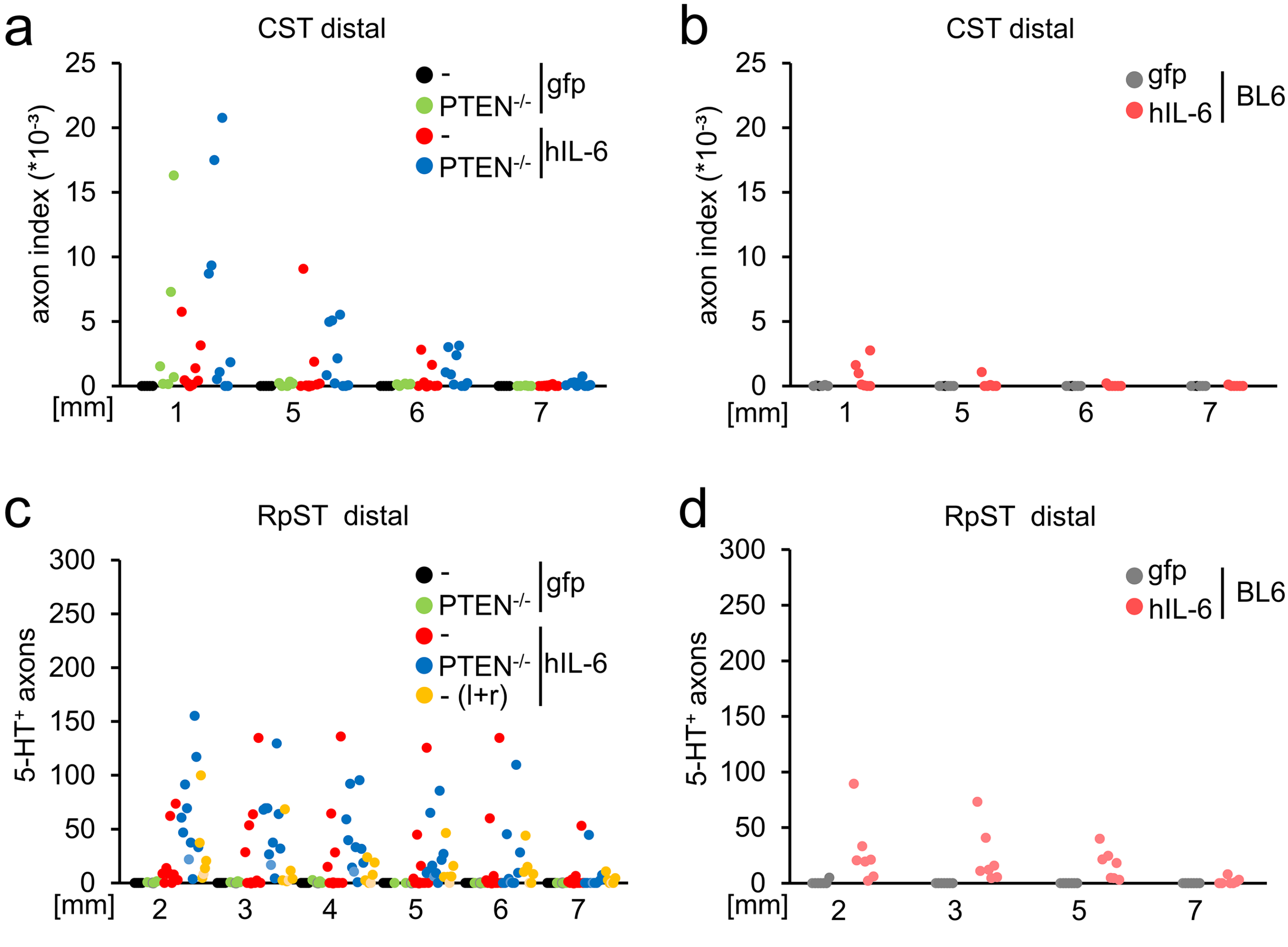

Supplementary Figure 4: Values of individual animals in CST and RpST axon regeneration

a/b) Axon index of regenerating CST axons from individual animals with an Ola background, as presented in **Fig. 2 i (a)** or BL6 animals, as presented in **Fig. S9 b** (**b**).

c/d) Quantification of regenerating serotonergic RpST axons from single animals with Ola background as presented in **Fig. 4 j** (**c**), or BL6 animals as presented in **Fig. S9 c** (**d**).

Source data are provided as a Source Data file.

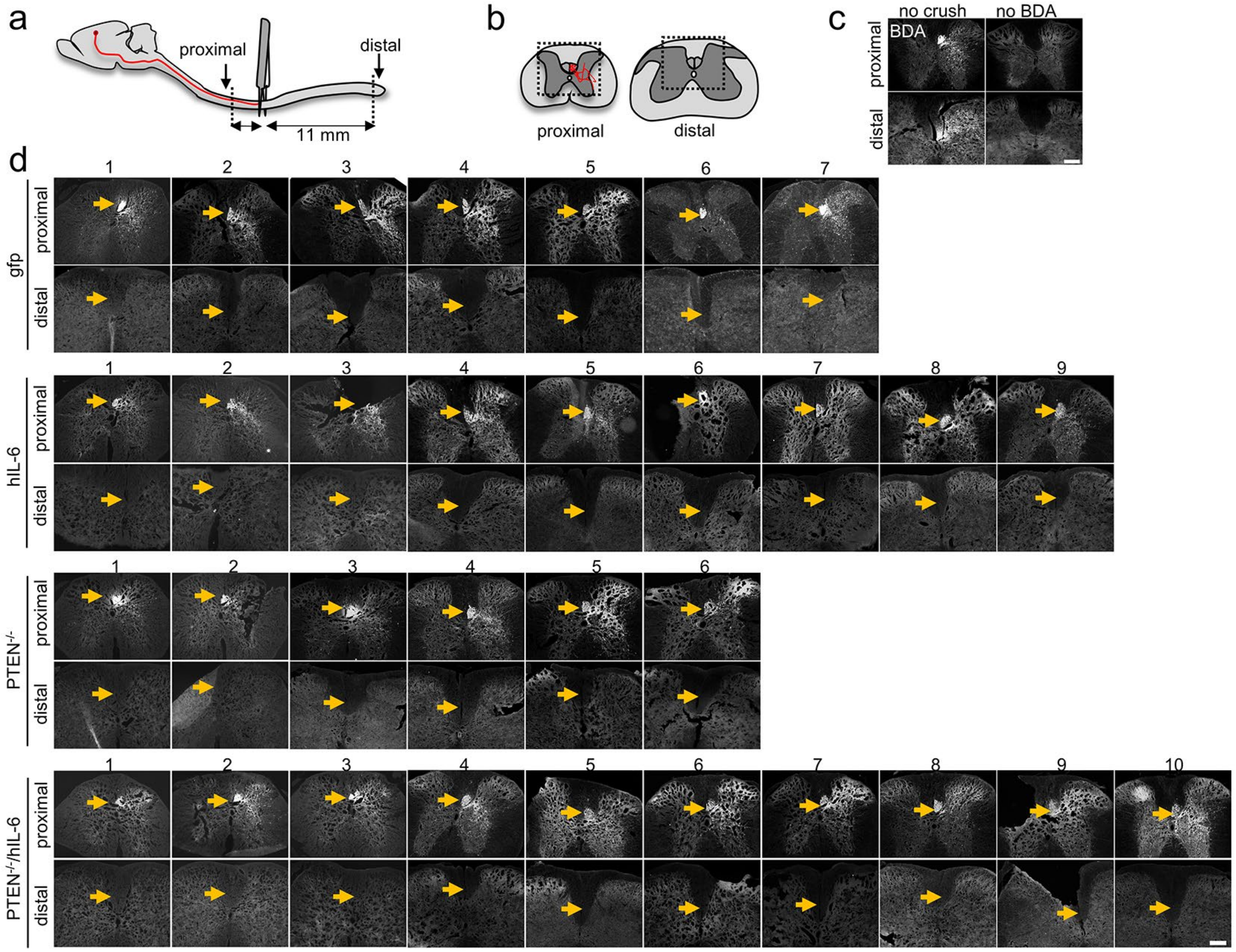

Supplementary Figure 5: Validation of complete spinal cord lesion

a) Schematic drawing illustrating the location of proximal and distal cross-sections used to analyze potential spared BDA-labeled axons in animals treated as described in **Fig. 2 a**.

b) Illustration of the expected location of BDA labeled CST axons (red) in proximal and distal cross-sections in case of the complete lesion without spared axons.

c) Coronal proximal and distal spinal cord cross-sections of an animal that had received cortical BDA injection but no spinal cord injury (no crush; positive control) and an animal without BDA injection (no BDA). Scale bar: 50 µm.

d) Images of proximal and distal spinal cord cross-sections of all animals, as described in **Fig. 2-4, Figs. S1, S4, S6**. Dotted boxes indicate imaged areas in **b**. Yellow arrows mark the CST. Scale bar: 50 µm.

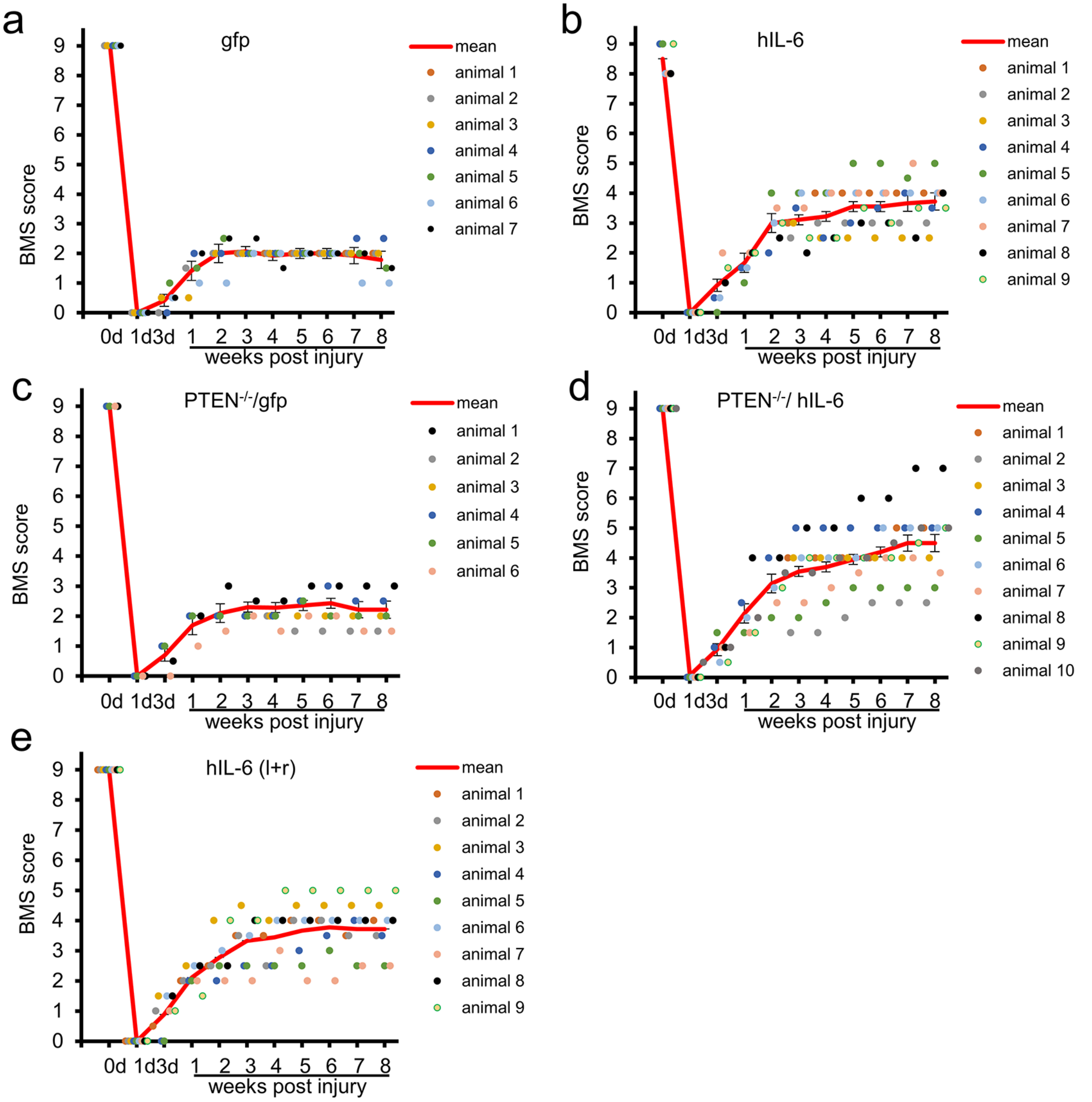

Supplementary Figure 6: BMS Values of individual animals in open field analysis

a-d) Average BMS scores of left and right hind paws from individual animals treated as described in **Fig. 3 a** and their respective mean values (red line), as presented in **Fig. 3 b**.

e) Average BMS scores of left and right hind paws and respective mean values (red line) from individual animals that received spinal cord crush and intracortical AAV2-hIL-6 injections in both hemispheres as presented in **Fig. 3 f**.

Source data are provided as a Source Data file.

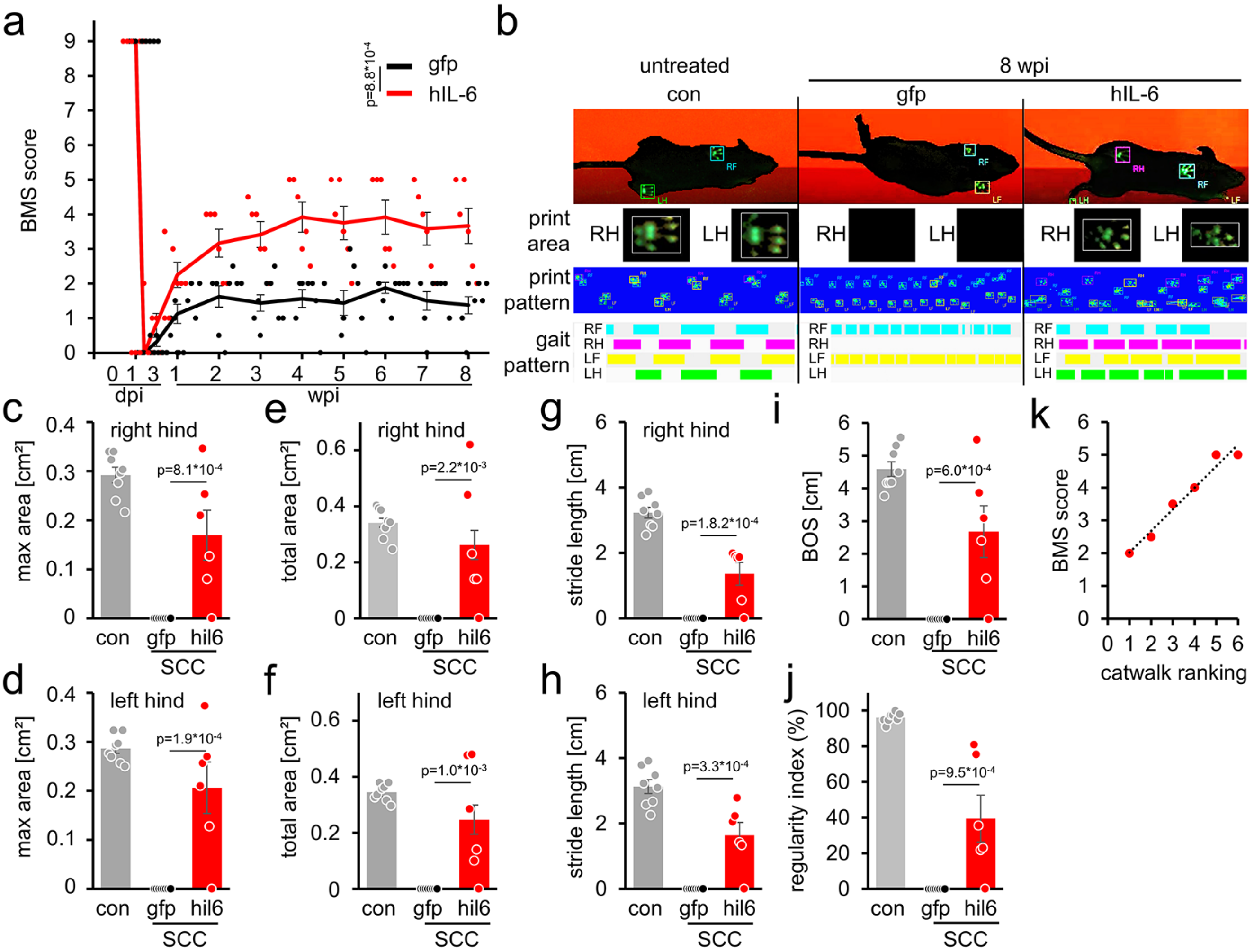

Supplementary Figure 7: Validation of functional recovery via *Catwalk* gait analysis

a) BMS score of mice received an intracortical injection of either AAV-gfp or AAV-hIL-6 after spinal cord crush. Animals were tested over 8 weeks post spinal cord injury (wpi). Values represent means +/- SEM of 6-8 animals per group (gfp, n=8; hIL-6 n=6), showing the left and right hind paw's average score.

b) Illustration of *Catwalk* analysis from mice as described in **a** and untreated controls (con), showing representative images of the maximal print area of left (LH) and right (RH) hind paws, print- and gait pattern (LF= left front paw, RF= right front paw; LH= left hind paw; RH= right hind paw). Catwalk gait analysis was performed after the final BMS testing 8 weeks post spinal cord crush.

c-i) *Catwalk* analysis of untreated controls and AAV-gfp- and AAV-hIL-6-treated animals after SCC: Evaluation of maximal contact (**c, d**) and total paw print area (**e, f**) during contact with the glass plate, stride length of both hind paws (**g, h**), and the base of support (BOS) reflected by the width between hind paw prints (**i**).

j) Catwalk analysis: Regularity index reflecting the degree of coordination between fore and hind paws in untreated controls and AAV-gfp or AAV-hIL6 tread mice after SCC.

k) Ranking of catwalk performance (from 1=worst performing to 6=best performing) of all tested hIL-6 treated mice as described in A based on all evaluated catwalk parameters shown in **c-j.** Data were plotted against the BMS score taken at 8 weeks post-injury (wpi) of the individual animals demonstrating linear correspondence between catwalk and BMS results.

Significances of intergroup differences were evaluated using the student's t-test **(a),** or one-way analysis respective p-values indicate of variance (ANOVA) with Tukey post hoc test (c-j).

Source data are provided as a Source Data file.

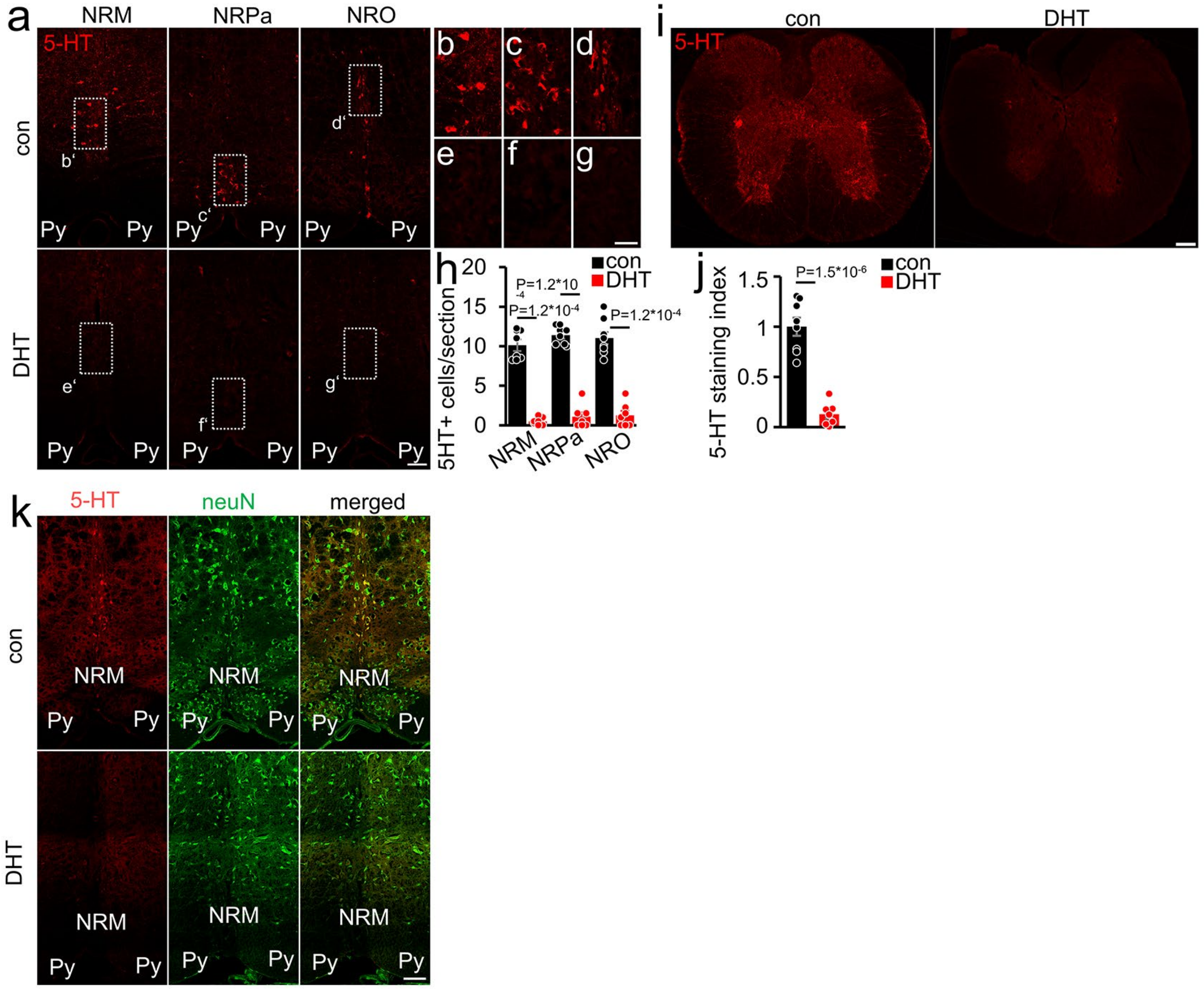

Supplementary Figure 8: Validation of DHT mediated depletion of raphe neurons

a) Images of raphe nuclei (NRM= nucleus raphe magnus, NRPa = nucleus raphe pallidus, NRO= nucleus raphe obscurus) located above the pyramidal tracts (Py) in coronal medullary sections from mice 1 day after intracerebroventricular 5,7-dihydroxytryptamine (DHT) injection, or untreated controls (con). Raphe neurons are visualized by serotonin (5-HT) immunostaining. Scale bar: 100 µm.

b-g) Higher magnification of indicated areas in images, as shown in **a**. Scale bar: 50 µm .

h) Quantification of serotonin (5-HT) positive raphe neurons per section in medullary immunostainings as described in **a**.

i) Images of thoracic spinal cord cross-sections from animals, as described in A. Raphe spinal axons are visualized by serotonin (5-HT) staining. Scale bar: 50 µm.

j) Quantification of 5-HT staining as sown in **i** for untreated (con, n=8) and DHT injected (DHT, n=7) mice.

k) Images of coronal medullary sections containing the NRM from mice 1 week after intracerebroventricular 5,7-dihydroxytryptamine (DHT) injection, or untreated controls (con). Raphe neurons are visualized by serotonin (5-HT) staining, and neuN staining shows all neurons. DHT specifically depleted serotonergic raphe but not other neurons.

Scale bar: 100 µm .

Significances of intergroup differences were evaluated using a one-way analysis of variance (ANOVA) with Tukey post hoc test **(h)** or the student's t-test **(j).** Respective p-values indicate significances.

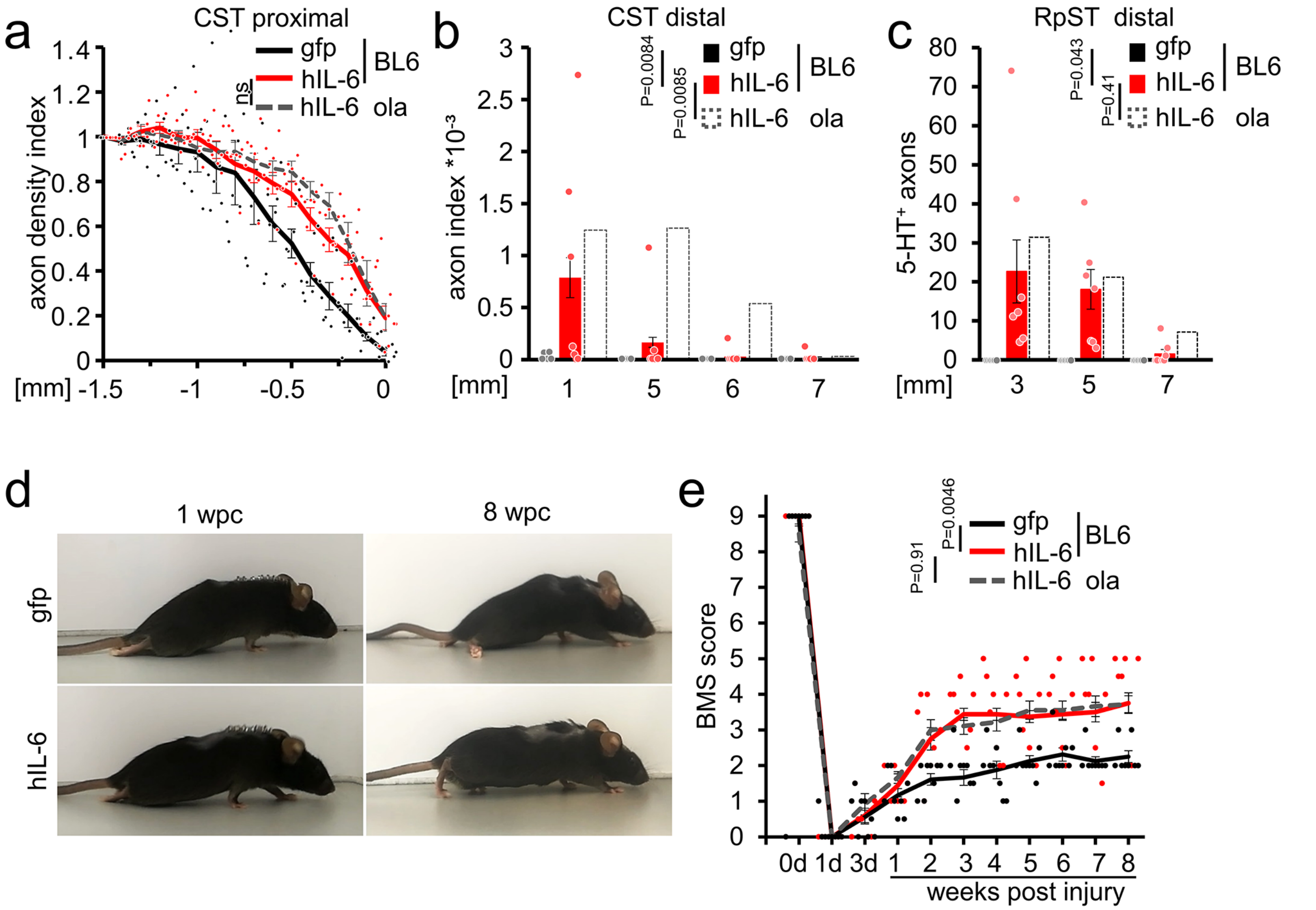

Supplementary Figure 9: Hyper-IL-6 promotes functional recovery in different mouse strains

a/b) Quantification of BDA-traced CST fibers proximal- (**a**), and regenerated axons distal (**b**) to the lesion site of non-transgenic BL6 mice 8 weeks after spinal cord crush (SCC). Animals received injections of either AAV2-GFP (gfp) or AAV2-hIL-6 (hIL-6) into the left sensorimotor cortex after SCC. The dashed line represents values of hIL-6 treated PTEN-floxed ($PTEN^{+/+}$) Ola mice from **Fig. 1** for comparison. Values represent means +/- SEM of 7-8 animals per group (gfp, n=7; hIL-6, n=8).

c) Regenerated 5-HT-positive RpST axons at indicated distances from the injury site in BL6 animals as described in **a**. Dashed bars represent values of hIL-6 treated PTEN-floxed ($PTEN^{+/+}$) Ola mice from **Fig. 4 j** for comparison. Values represent means +/- SEM of 7-8 animals per group (gfp, n=7; hIL-6, n=8).

d) Representative pictures showing the open-field movement of non-transgenic BL6 mice as described in **a** 1 and 8 weeks after SCC.

e) BMS score of animals as described in **a** at indicated time points after SCC. Dots represent individual animals' values from the gfp (black) and the hIL-6 (red) group. The dashed gray line shows values of AAV2-hIL-6-treated PTEN-floxed ($PTEN^{+/+}$) Ola animals from **Fig. 3 b** for comparison. Values represent means +/- SEM of 8-9 animals per group (gfp, n=8; hIL-6, n=9), showing the left and right hind paws' average score. Significances of intergroup differences were evaluated in **a, b, c,** and **e** (at 8 weeks post-injury) using a one-way analysis of variance (ANOVA) with Tukey or Holm-Sidak post hoc tests. P-values indicate statistical significance. ns=non-significant.

Source data are provided as a Source Data file.

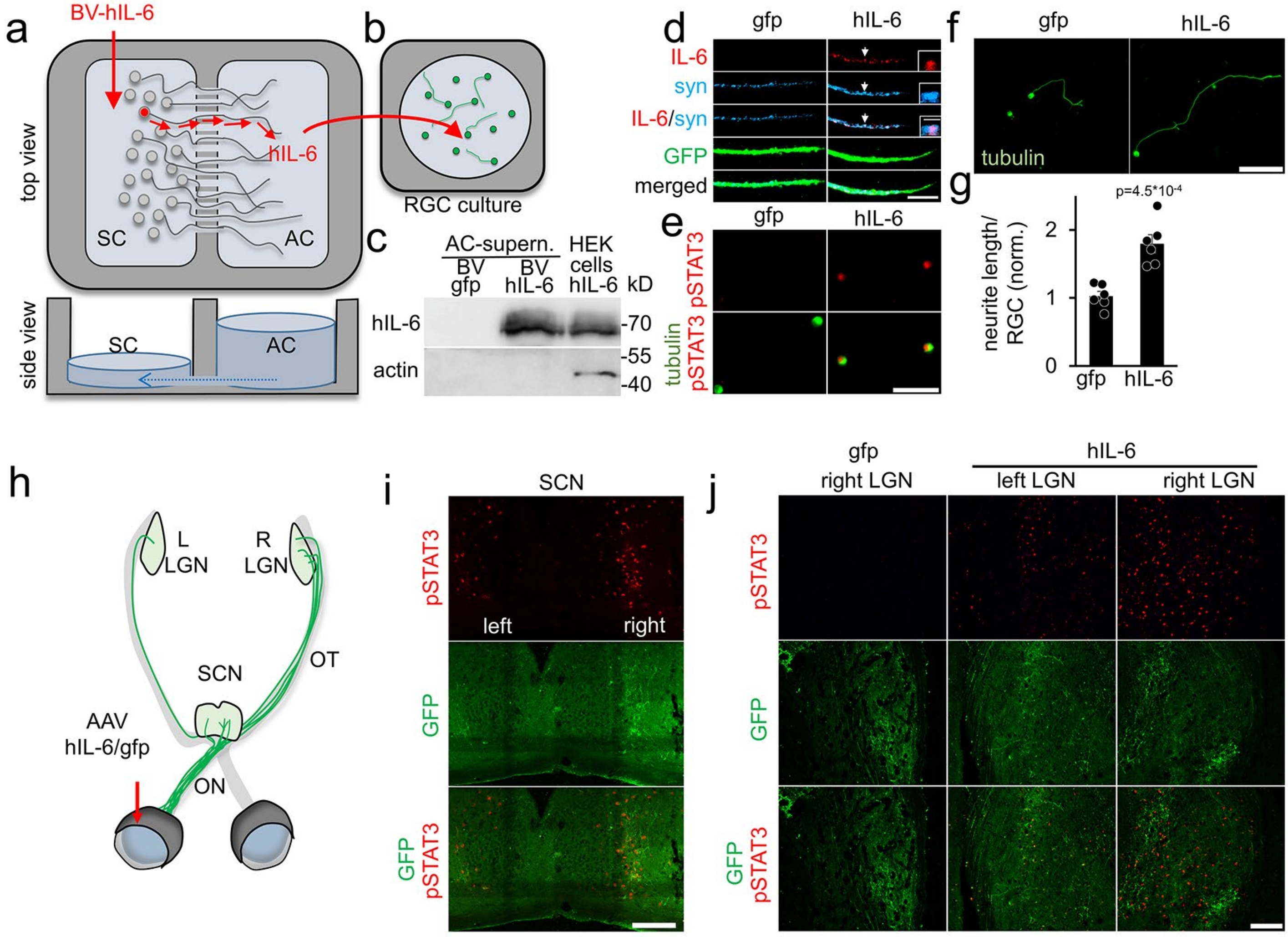

Supplementary Figure 10: Axonal transport and release of virally expressed hIL-6

a/b) Schematic illustration of the experiment shown in **c-g**. Primary adult dorsal root ganglion-(DRG) neurons were cultured in microfluidic two-compartment chambers. Cell bodies in the soma compartment (SC) were transduced with a baculovirus expressing hIL-6 (BV-hIL-6) or with a GFP expressing control virus (BV-GFP). The diffusion of molecules from the SC to the axonal compartment (AC) was prevented by an antagonistic microflow (blue dotted arrow) due to different volumes of the medium in the AC and SC. Hyper-IL-6 is transported within axons of transduced neurons and released at axon terminals in the AC **(a)**. After 48 hours, media from AC of BV-GFP or BV-hIL-6 treated cultures were collected and used for culturing primary retinal ganglion cells (RGCs) **(b)**.

c) Western blot analysis of the supernatant from the axonal compartment (AC-supern.) of cell cultures as described in A, showing the presence of hIL-6 after BV-hIL-6 but not BV-GFP treatment. Lysates of HEK293 cells transduced with BV-hIL-6 were used as a positive control. The lack of β-actin in the AC-supernatant proves the absence of cell fragments in the lysate, indicating the release of hIL-6 into the medium.

d) Representative images of GFP (green) stained axonal tips from BV-gfp (gfp) or BV-hIL-6 (hIL-6) treated cultured DRG neurons as described in **a** stained for hIL-6 (IL-6, red) and synapsin (blue). Hyper-IL-6-positive vesicles were detected in only in axons of BV-hIL-6 treated neurons. The white arrow indicates an area magnified in insets. Scale bar: 10 µm, insets: 2 µm.

e) Representative images showing phospho-STAT3 (pSTAT3, red) in nuclei of βIII-tubulin (tubulin, green) positive RGCs 1 day after culturing in medium derived from BV-hIL-6 (hIL-6) treated DRG neuron cultures as described in **a, b**. Medium from BV-gfp (gfp) revealed no STAT3 activation. Scale bar: 50 µm.

f) Representative images of neurite growth in βIII-tubulin (tubulin, green) stained RGCs as described in **a/b/d** exposed to hIL-6 or GFP-conditioned media. Scale bar: 100 µm.

g) Quantification of neurite length per RGC in retinal cultures as depicted in E. Data represent means ± SEM of six independent experiments (n=6), each with four wells as technical replicate per treatment. Significances of intergroup differences were evaluated using Student's t-test as indicated by p values.

h) Illustration of the mouse visual pathway with the optic nerves (ON), optic tracts (OT), the suprachiasmatic nucleus (SCN), and lateral geniculate nuclei (LGN). 95% of transduced RGCs (green) of the left eye project axons into the right LGN; only 5% into the left hemisphere. The red arrow indicates intravitreal AAV2-hIL-6 injection.

i) The coronal section of both hemispheres of the SCN immunohistochemically stained for phosphorylated STAT3 (pSTAT3, red) from mice 3 weeks after intravitreal AAV2-hIL-6 injection. Staining was detected near GFP-positive axons of AAV2-hIL-6 transduced RGCs and was predominantly in the right hemisphere. Scale bar: 100 µm.

j) Coronal sections of the left and right LGN 3 weeks after intravitreal injection of either AAV2-hIL-6 (hIL-6) or AAV2-GFP (gfp) into the left eyes. Phospho-STAT3 (pSTAT3, red) was only detected in hIL-6-treated animals near GFP-positive axons (green) predominantly in the right hemisphere. Scale bar: 100 µm. Significances of intergroup differences in **g** were evaluated using Student's t-test as indicated by p-values.

Source data are provided as a Source Data file.

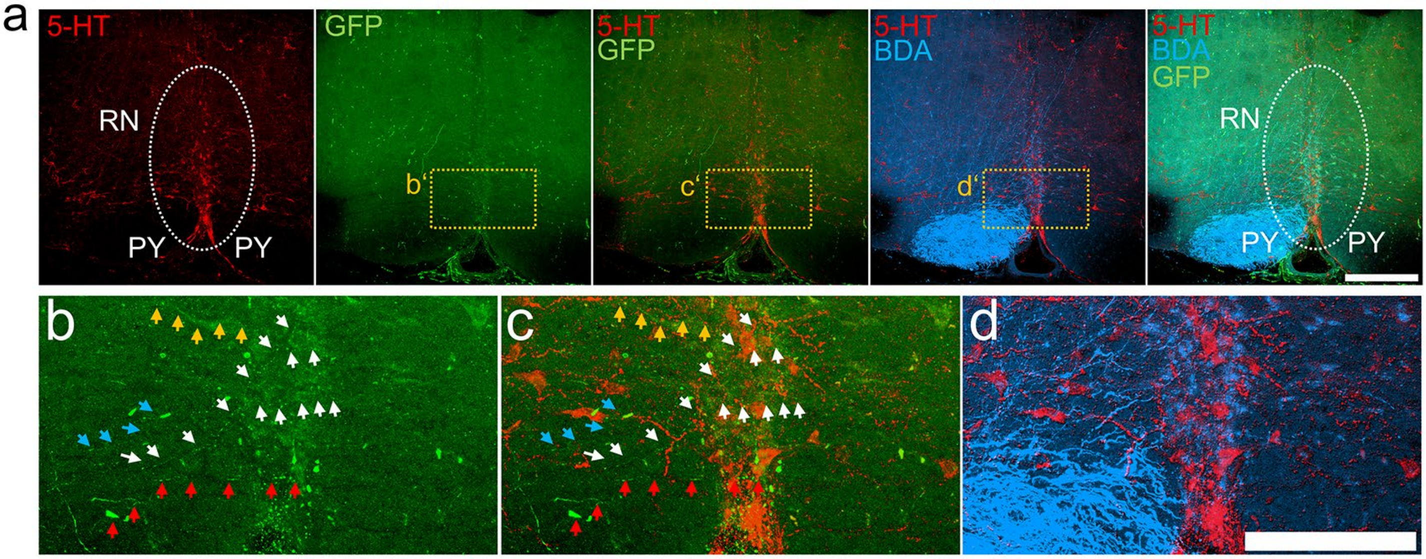

Supplementary Figure 11: CST axon collaterals project to raphe nuclei

a) Maximum intensity projection of coronal scan through 50 µm of cleared brain stem tissue from mice subjected to SCC and AAV2-hIL-6 injection as described in **Fig. 1 a:** BDA-traced pyramidal (PY) CST axons (blue) and serotonergic neurons of the raphe nuclei (RN) were visualized by 5-HT immunostaining (red). Axons of AAV2-hIL-6 transduced CST neurons were visualized by GFP co-expression (green). Scale bar: 100 µm

b-d) Higher magnification of dashed boxes, as presented in **a**. Arrows of different colors follow respective paths of individual GFP-positive axons and their collaterals projecting to the raphe nucleus. Scale bar: 100 µm.

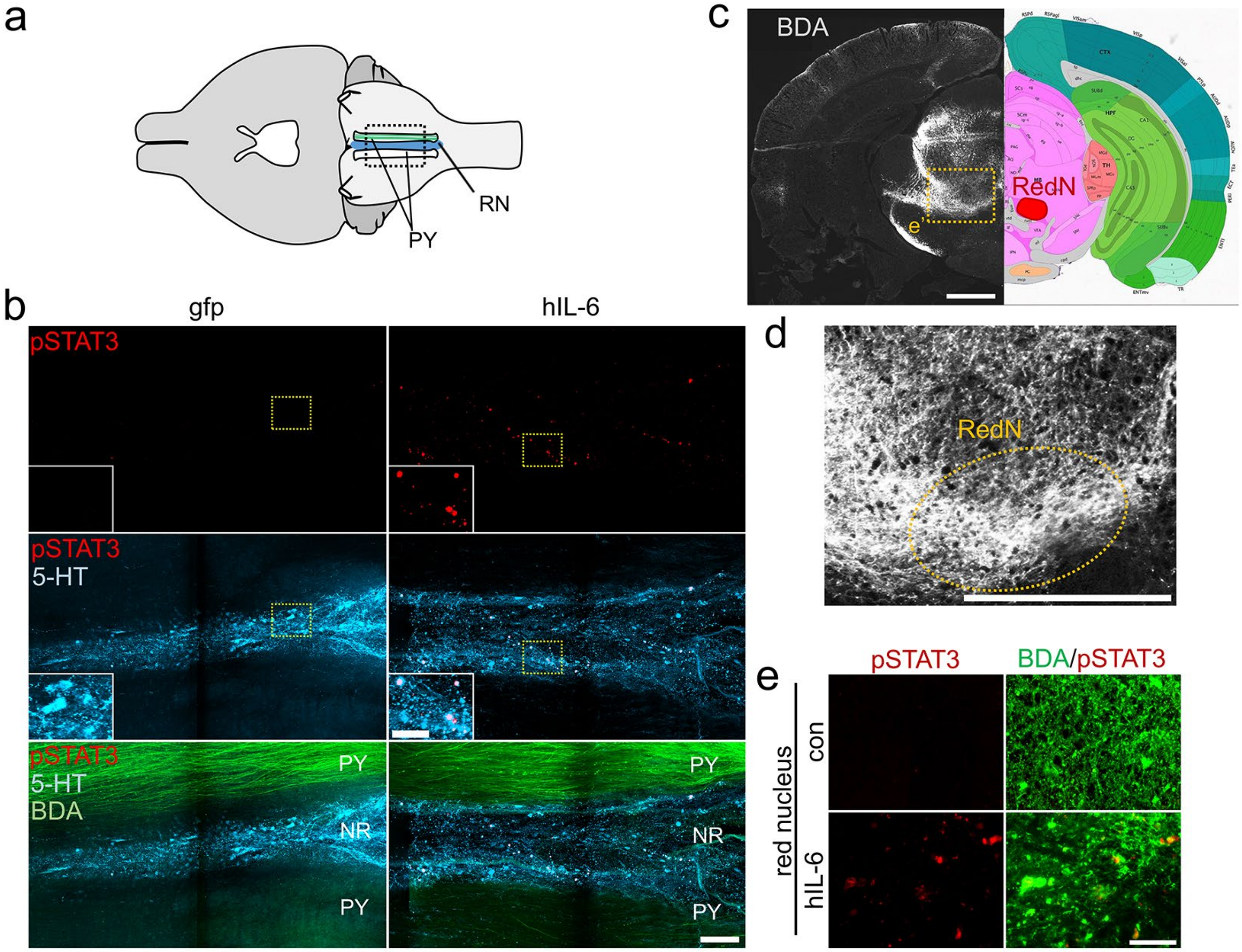

Supplementary Figure 12: Hyper-IL-6 transneuronally stimulates neurons of raphe and red nuclei

a) Schematic illustration related to images shown in B**:** Bottom view of the mouse brain showing pyramidal tracts (PY) and raphe nuclei (RN, blue) in the medial column of the medulla. Axons of GFP-expressing transduced cortical neurons in the left pyramid are indicated in green. The dotted box illustrates the area used for confocal scans from cleared tissue, as shown in **b**.

b) Maximum intensity projections of transverse confocal scans through 150 µm of cleared brain tissue as depicted in **a** from mice after unilateral (left) intracortical injections of AAV2-GFP (gfp) or AAV2-hIL-6 (hIL-6) and BDA treatment. Before tissue clearing, brains were stained against serotonin (5-HT, blue) and phosphorylated STAT3 (pSTAT3, red), while BDA labeled CST axons were visualized using fluorescently labeled streptavidin (BDA, green). Insets represent higher magnifications of areas in dashed yellow boxes. Scale bar: 50 µm; 25 µm for insets.

c) Coronal midbrain section 2 weeks after intracortical BDA injection. BDA labeled axons of cortical motor neurons project into the red nucleus (RedN). Localization of the RedN is indicated in red in a corresponding map from Allen Brain Atlas (left). Scale bar: 500 µm.

d) Higher magnification of the dashed box shown in **c**. Scale bar: 500 µm

e) Images of pSTAT3 (red) immunostained coronal sections of the ipsilateral red nucleus 3 weeks after intracortical injection with either AAV2-GFP (con) or AAAV2-hIL-6 (hIL-6). RedN cell bodies were visualized by BDA (green) after direct injection into the red nucleus using specific coordinates 2 weeks before tissue harvest. Phospho-STAT3 is present in neurons of the RedN only after cortical AAV2-hIL-6 but not AAV2-GFP treatment. Scale bar: 50 µm

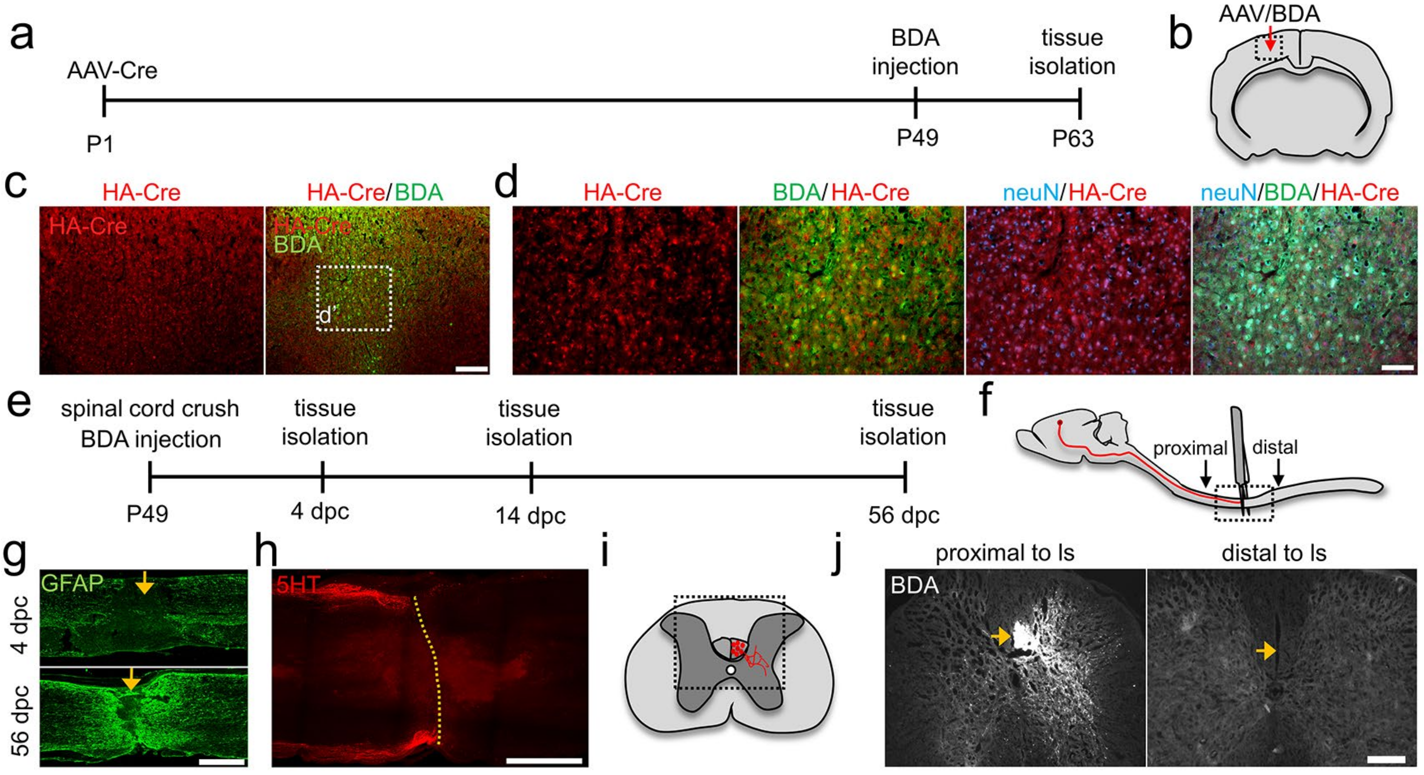

Supplementary Figure 13: Validation of intracortical AAV2-application and spinal cord crush

a) Timeline for intracortical AAV2-HA-Cre (AAV2-Cre) treatment starting at postnatal day 1 (P1), BDA tracing of corticospinal neurons (P49), and tissue isolation (P63).

b) Schematic drawing illustrates the virus and BDA injection site (arrow) in the sensorimotor cortex. The dashed box indicates the location of images presented in **c.**

c) The coronal section of the sensorimotor cortex was stained for the HA-Cre (red) and BDA (green). The dashed box indicates the magnified area in **d**. Scale bar: 200 µm.

d) Higher magnification of the image shown in **c,** including NeuN staining (blue). Ca. 80% of BDA-positive neurons in layer V of the sensorimotor cortex were also HA-Cre-positive. Scale bar: 50 µm.

e) Timeline for experiments verifying the completeness of spinal cord crush (SCC). P49 mice were subjected to SCC, and tissues isolated 4, 14, or 56 days after spinal cord crush (dpc).

f) Schematic drawing illustrating BDA tracing in the CST at the thoracic vertebra 8 (T8) and the SCC level. The dashed box indicates the location of the immunohistochemical sections presented in **g and j**.

g) Sagittal sections of thoracic spinal cord stained for GFAP (green) 4 and 56 dpc. Lack of astrocytes at the lesion site at 4 dpc verifies the completeness of the lesion. Eight weeks after crush, astrocytes bridged the lesion site. Scale bar: 500 µm.

h) Maximum intensity projection of a confocal scan with a z-range of 500 µm from dorsal to ventral through a cleared spinal cord 4 dpc after prior serotonin (5-HT, red) immunostaining. Scale bar: 500 µm.

i) Schematic drawing illustrating the location (dashed box) of images in **j** in spinal cord cross-sections. Labeled CST axons are indicated in red.

j) Coronal sections of the spinal cord 2 weeks after SCC and intracortical BDA injection showing traced CST axons (arrow) proximal, but not distal to the lesion site (ls). Scale bar: 250 µm.

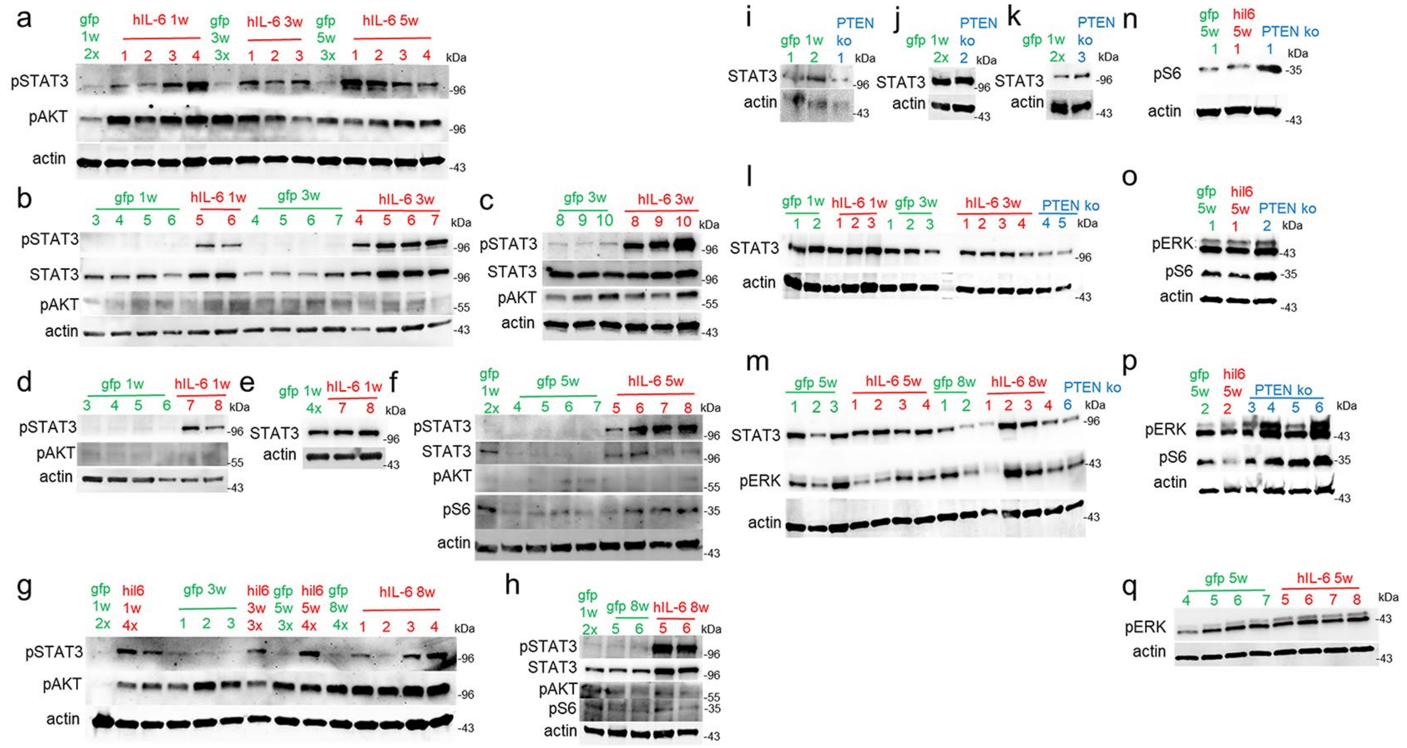

Supplementary Figure 14: Western blots used for analysis in Fig. 1

a-m) Western blots of single cortical lysates from each animal analyzed in Fig. 1 O-Q. Treatment groups, as described in Fig. 1. Samples were either loaded individually or mixed to equal proportions from 2-4 different animals of the same group (2x- 4x). Lysates from individual mice are labeled with numbers from 1-10.

m-q) Western blots of single cortical lysates from each animal analyzed in Fig. **1 s** and **t**. Treatment groups, as described in Fig. 1.

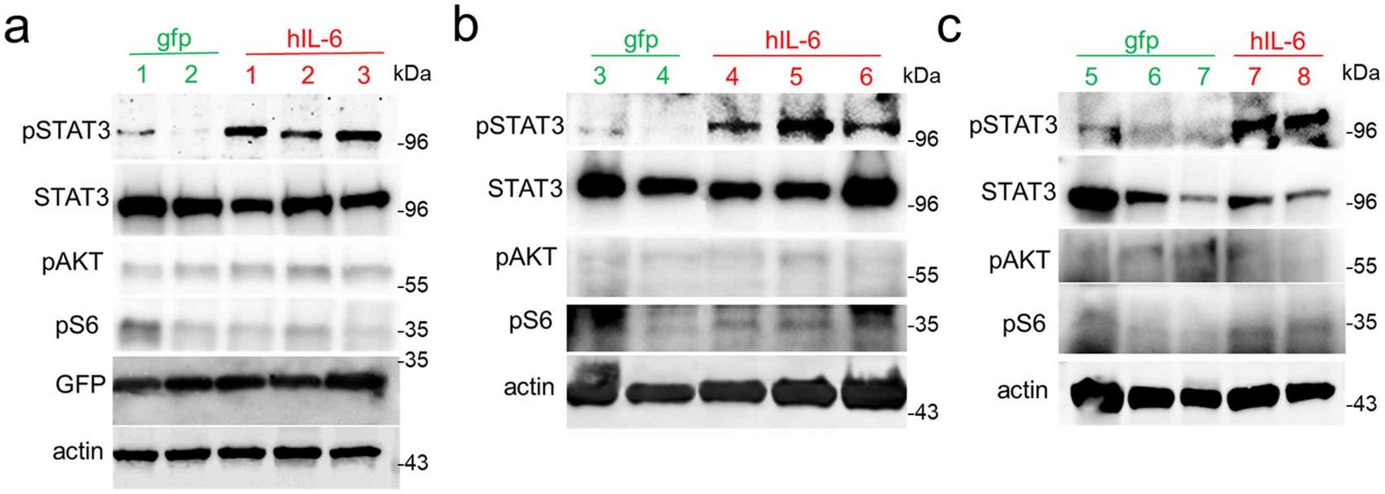

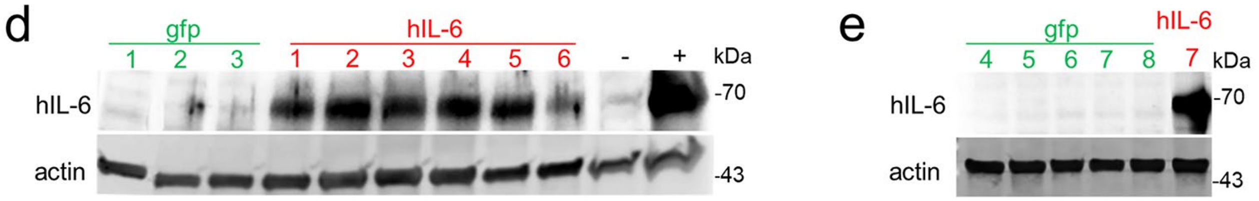

Supplementary Figure 15: Western blots used for analysis in Fig. 6 and Fig. S1

a-c) Western blots of single cortical lysates from each animal analyzed in **Fig. 6c**. Samples are labeled with numbers from 1-8 for individual mice. Treatment groups, as described in Fig. 6.

d/e) Western blots of single cortical lysates from each animal analyzed in Fig. **S1 i**. Treatment groups, as described in **Fig. S1**. In D, negative and positive controls of HEK293 cell lysates transfected with either GFP (-) or hIL-6 (+) plasmids were included.

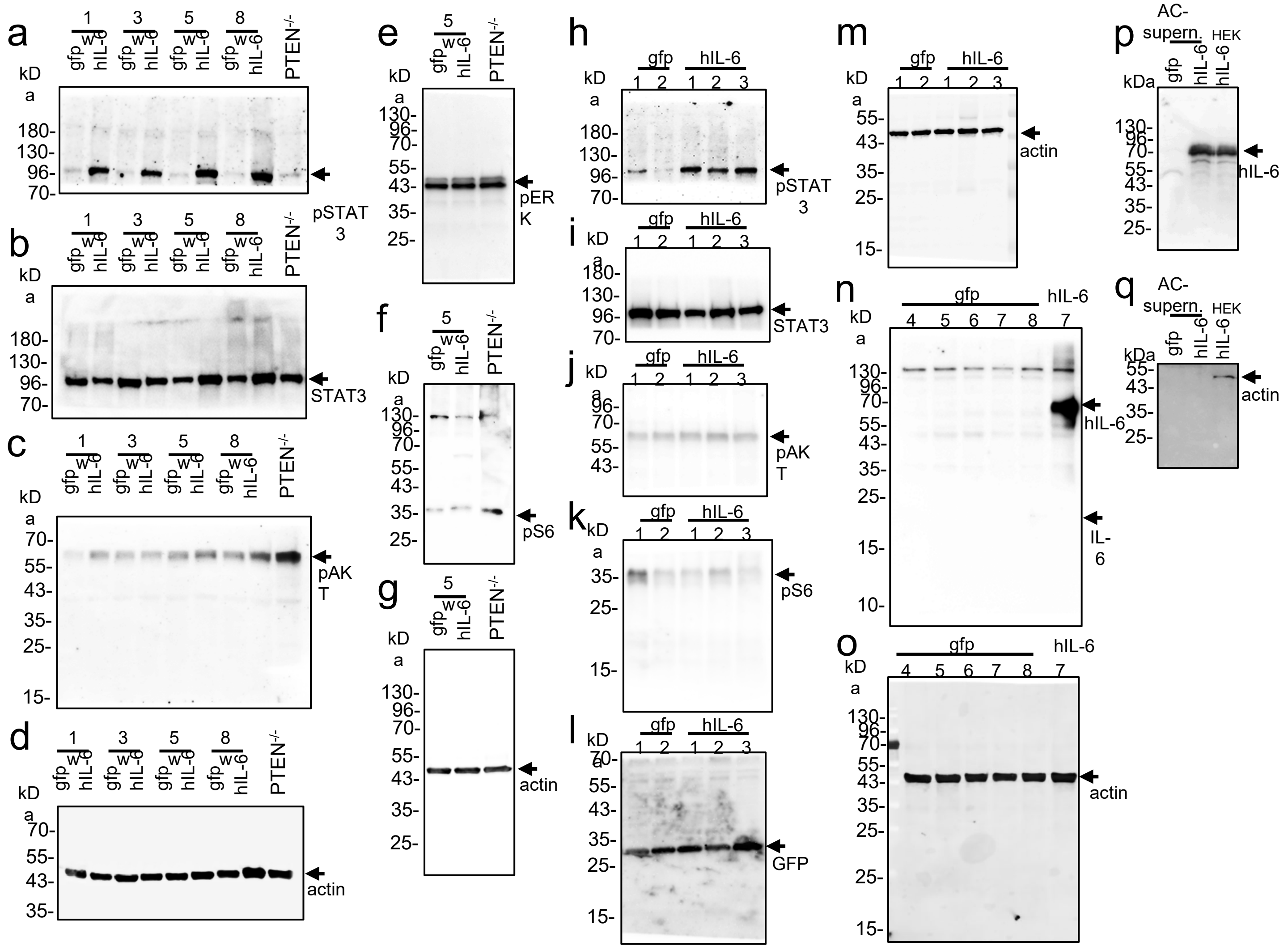

Supplementary Figure 16: Membranes from western blots shown in Fig.1n, r; Fig. 6b, Fig. S1h, Fig. S10c

a-d) Whole images of Western blot membranes as shown in Fig. 1n.

e-g) Whole images of Western blot membranes as shown in Fig.1r.

h-m) Whole images of Western blot membranes as shown in Fig. 6c.

n-o) Whole images of Western blot membranes as shown in Fig. S1h.

p-q) Whole images of Western blot membranes as shown in Fig. S10c.
