# Supplementary material for: Transneuronal delivery of hyper-interleukin-6 enables functional recovery after severe spinal cord injury in mice
Source: Nat Commun. 2021 Jan 15;12:391. doi: 10.1038/s41467-020-20112-4 (PMC7810685; doi:10.1038/s41467-020-20112-4)
Supplement: Supplementary file 3 — Description of Additional Supplementary Files [file 41467_2020_20112_MOESM3_ESM.pdf]

Description of Additional Supplementary Files

Supplementary Movie 1: Open field locomotion after SCC and AAV2- GFP treatment The video shows a PTEN-floxed Ola mouse (PTEN+/+) that received an injection of AAV2- GFP into the left sensorimotor cortex at postnatal day 1. After 7 weeks, the mouse was subjected to complete spinal cord crush (SCC) (T8) and subsequently received an intracortical (left) injection of AAV2-GFP (see Fig. 1a). Videos were recorded at 1 and 8 weeks and SCC.

Supplementary Movie 2: Open field locomotion after PTEN-/- and SCC The video shows a PTEN-floxed Ola mouse that received an injection of AAV2-Cre (PTEN-/-) into the left sensorimotor cortex at postnatal day 1. After 7 weeks, the mouse was subjected to severe spinal cord crush (SCC) (T8) and subsequently received an intracortical (left) injection of AAV2-GFP (see Fig. 1a). Videos were recorded at 1 and 8 weeks after SCC.

Supplementary Movie 3: Open field locomotion after SCC and AAV2-hIL-6 treatment The video shows a PTEN-floxed Ola mouse (PTEN+/+) that received an injection of AAV2- GFP into the left sensorimotor cortex at postnatal day 1. After 7 weeks, the mouse was subjected to severe spinal cord crush (SCC) (T8) and subsequently received an intracortical (left) injection of AAV2-hIL-6 (see Fig. 1 a). Videos were recorded at 1 and 8 weeks after SCC.

Supplementary Movie 4: Open field locomotion after PTEN-/- and SCC with AAV2-hIL-6 treatment The video shows a PTEN-floxed Ola mouse that received an injection of AAV2-Cre (PTEN-/-) into the left sensorimotor cortex at postnatal day 1. After 7 weeks, the mouse was subjected to severe spinal cord crush (SCC) (T8) and subsequently received an intracortical (left) injection of AAV2-hIL-6 (see Fig. 1a). Videos were recorded at 1 and 8 weeks and SCC. - 3 –

Supplementary Movie 5: Open field locomotion and Catwalk gait analysis determined in mice after SCC and intracortical AAV2-GFP, or AAV2-hIL-6 treatment The video shows two wild-type mice subjected to severe spinal cord crush (SCC) (T8) and subsequently received an intracortical injection of AAV2-GFP or AAV2-hIL-6 into the left sensorimotor cortex. Videos were recorded at 1day and 8 weeks post SCC. Additionally, Catwalk analysis was recorded after 8 weeks after SCC.

Supplementary Movie 6: Open field locomotion after DHT- mediated depletion of raphe spinal input The video shows a PTEN-floxed Ola mouse (PTEN+/+) that was subjected to severe spinal cord crush (SCC) (T8) and subsequently received intracortical injections of AAV2-hIL-6. To abolish raphe spinal input, the animal was injected intracerebroventricularly with the serotonin neurotoxin 5,7-dihydroxytryptamine (DHT) 6 weeks after SCC. Videos were recorded at 1 and 6 weeks after SCC and

1 day and 1 week after DHT application. Additionally, another mouse of the same background that received a similar treatment but was injected with AAV2-GFP instead of AAV-hIL-6 was recorded 6 weeks after SCC and 1 day after DHT treatment.

Supplementary Movie 7: Open field locomotion determined in a BL6 mouse after SCC and AAV2-hIL-6 treatment T he video shows a C57BL/6J wildtype mouse (BL6) that was subjected to severe spinal cord crush (SCC) (T8) and subsequently received an intracortical injection of AAV2-hIL-6 into the left sensorimotor cortex. Videos were recorded at 1 and 8 weeks after SCC. - 4 –

Supplementary Movie 8: Intracortical hIL-6 application induces STAT3 phosphorylation in raphe nuclei Three-dimensional projection of transverse confocal scan through 150 µm of cleared brain stem tissue after unilateral (left) intracortical injection of AAV2-hIL-6 and BDA treatment. Serotonin (5-HT) was stained in blue, phosphorylated STAT3 (pSTAT3) in red, and BDA labeled CST axons in green. Localization of the imaged area and a 2D projection image, including a scale bar, are presented in Fig. S12 a, b.
