# Supplementary material for: Transneuronal delivery of hyper-interleukin-6 enables functional recovery after severe spinal cord injury in mice
Source: Nat Commun. 2021 Jan 15;12:391. doi: 10.1038/s41467-020-20112-4 (PMC7810685; doi:10.1038/s41467-020-20112-4)
Supplement: Supplementary file 12 — Reporting Summary [file 41467_2020_20112_MOESM12_ESM.pdf]

nature research

Corresponding author(s): Dietmar Fischer

Last updated by author(s): Sep 9, 2020

Reporting Summary

Nature Research wishes to improve the reproducibility of the work that we publish. This form provides structure for consistency and transparency in reporting. For further information on Nature Research policies, see Authors & Referees and the Editorial Policy Checklist .

Statistics

For all statistical analyses, confirm that the following items are present in the figure legend, table legend, main text, or Methods section.

n/a	Confirmed	
☐	☒	The exact sample size (*n*) for each experimental group/condition, given as a discrete number and unit of measurement
☐	☒	A statement on whether measurements were taken from distinct samples or whether the same sample was measured repeatedly
☐	☒	The statistical test(s) used AND whether they are one- or two-sided<br>*Only common tests should be described solely by name; describe more complex techniques in the Methods section.*
☒	☐	A description of all covariates tested
☐	☒	A description of any assumptions or corrections, such as tests of normality and adjustment for multiple comparisons
☐	☒	A full description of the statistical parameters including central tendency (e.g. means) or other basic estimates (e.g. regression coefficient) AND variation (e.g. standard deviation) or associated estimates of uncertainty (e.g. confidence intervals)
☐	☒	For null hypothesis testing, the test statistic (e.g. *F*, *t*, *r*) with confidence intervals, effect sizes, degrees of freedom and *P* value noted<br>*Give P values as exact values whenever suitable.*
☒	☐	For Bayesian analysis, information on the choice of priors and Markov chain Monte Carlo settings
☒	☐	For hierarchical and complex designs, identification of the appropriate level for tests and full reporting of outcomes
☒	☐	Estimates of effect sizes (e.g. Cohen's *d*, Pearson's *r*), indicating how they were calculated

Our web collection on statistics for biologists contains articles on many of the points above.

Software and code

Policy information about availability of computer code

Data collection	Axiovision 4.9 Zeiss; LASX 3.5.1 Leica; Virtual Slide 2.9 Olympus, ZEN 2.3 Zeiss; Catwalk XT 10.6 Noldus
Data analysis	Sigma STAT 3.2 Systat Software; ImageJ 1.51f (https://imagej.nih.gov/ij/); Adobe Photoshop CS5 version 12; Catwalk XT 10.6 Noldus

For manuscripts utilizing custom algorithms or software that are central to the research but not yet described in published literature, software must be made available to editors/reviewers. We strongly encourage code deposition in a community repository (e.g. GitHub). See the Nature Research guidelines for submitting code & software for further information.

Data

Policy information about availability of data

All manuscripts must include a data availability statement. This statement should provide the following information, where applicable:

- Accession codes, unique identifiers, or web links for publicly available datasets
- A list of figures that have associated raw data
- A description of any restrictions on data availability

Data Availability Statement
All data generated or analysed during this study are included in this manuscript (and its supplementary information files).

Field-specific reporting

Please select the one below that is the best fit for your research. If you are not sure, read the appropriate sections before making your selection.

☒ Life sciences ☐ Behavioural & social sciences ☐ Ecological, evolutionary & environmental sciences

For a reference copy of the document with all sections, see nature.com/documents/nr-reporting-summary-flat.pdf

Life sciences study design

All studies must disclose on these points even when the disclosure is negative.

Sample size	The sample size was calculated using Sigma STAT3.2 software with a power of 0.8, alpha of 0.05, minimum detectable difference of 0.25 and expected standard deviation of 0.1.
Data exclusions	Only animals with incomplete spinal cord crus due to technical issues during surgery were not considered for evaluation. Incomplete crush was indicated by a BMS score > 0 one day after surgury and the presence of spared axons > 10 mm caudal to the lesion site. Exclusion criteria were pre-establishe. Two animals with PTEN-/- and one control mouse that had received AAV-GFP were excluded because of not meeting the criteria described above. This refers to Data from Fig. 2, 3, and 4.
Replication	In vivo regeneration, functional recovery western blot and immunohistochemistry data were repeated 4-6 times with 6-10 independent samples from individual animals in total. All data were reproducible.
Randomization	Male and female littermates were randomly and allocated to experimental groups.
Blinding	Investigators were blinded during data collection and analysis.

Reporting for specific materials, systems and methods

We require information from authors about some types of materials, experimental systems and methods used in many studies. Here, indicate whether each material, system or method listed is relevant to your study. If you are not sure if a list item applies to your research, read the appropriate section before selecting a response.

Materials & experimental systems

n/a	Involved in the study	
☐	☒	Antibodies
☒	☐	Eukaryotic cell lines
☒	☐	Palaeontology
☐	☒	Animals and other organisms
☒	☐	Human research participants
☒	☐	Clinical data

Methods

n/a	Involved in the study	
☒	☐	ChIP-seq
☒	☐	Flow cytometry
☒	☐	MRI-based neuroimaging

Antibodies

Antibodies used	synapsin rabbit polyclonal, Millipore, Cat# AB1543 (RRID:AB_2200400), pERK1/2 polyclonal rabbit cell signaling technology Cat# 9101(RRID: AB_331646), GFAP polyclonal rabbit, Santa Cruz Biotechnology, Cat# sc-33673 (RRID: AB_627673); β-actin mouse monoclonal, Cat# A5441 (RRID: AB_476744); anti-mouse, anti- goat and anti-rabbit conjugated to Alexa Fluor 405, 488, 594, Invitorgen Cat# A21203, A-21207, A21206, A-21202; anti- goat, anti- mouse and anti-rabbit conjugated to Alexa Fluor 405 Jackson ImmunoResearch Cat# 711-475-152, 715-475-150, 705-475-147, ;anti-rabbit HRP conjugated, Cell Signaling Technology, Cat# 5127 (RRID: AB_10892860); anti-mouse IgG conjugated HRP; pSTAT3 monoclonal, rabbit, Cell Signalling Technology,Cat# 9145 (RRID:AB_2491009); STAT3 polyclonal, rabbit, Cell Signalling Technology,Cat# 12640 (RRID:AB_2629499); Cd11b, rat monoclonal, Bio-Rad, Cat# MCA74GA (RRID:AB_324660); IL-6 polyclonal, rabbit, Abcam, Cat# ab6672 (RRID:AB_2127460); polyclonal HA antibody,Sigma-Aldrich, Cat# H6908 (RRID:AB_260070); polyclonal GFP antibody, Novus, Cat# NB100-1770 (RRID:AB_10128178); pS6 polyclonal rabbit, Cell Signaling Technology Cat# 4857 (RRID:AB_2181035); βIII-tubulin monoclonal mouse, Covance Cat# MMS-435P (RRID:AB_2313773); T308-phosphorylated AKT, Cell Signaling Technology Cat# 13038 (RRID:AB_2629447), neuN, monoclonal mouse, Abcam Cat# ab177487, (RRID:AB_2532109); 5-HT (serotonin), polyclonal rabbit, goat antibodies ImmunoStar Cat# 20079, Cat# 20080 (RRID:AB_572262, RRID:AB_572263)
Validation	β-actin mouse monoclonal (RRID: AB_476744)<br>Target:<br>slightly modified β-cytoplasmic actin N-terminal peptide, Ac-Asp-Asp-Asp-Ile-Ala-Ala-Leu-Val-Ile-Asp-Asn-Gly-Ser-Gly-Lys, conjugated to KLH pig, hirudo medicinalis, bovine, rat, canine, feline, human, rabbit, carp, mouse, guinea pig, chicken, sheep<br>Validation:<br>According to the manufacturers homepage<br>Publications:<br>PMID:18785628, PMID:19399893, PMID:20653035, PMID:21456011, PMID:21491429, PMID:23322532, PMID:23515289, PMID:23515291, PMID:23568554, PMID:23677932, PMID:23751871, PMID:23766131, PMID:23782942, PMID:23904355, PMID:24141994, PMID:24169559, PMID:24274985, PMID:24424044, PMID:24428528, PMID:24428531, PMID:24601881, PMID:24885573, PMID:24926823, PMID:24990918, PMID:25319692, PMID:25416145, PMID:25474204, PMID:25556631, PMID:25562616, PMID:25621564, PMID:25679868, PMID:25849727, PMID:26004679, PMID:26237551, PMID:26251011, PMID:26295369, PMID:26562260, PMID:26568405, PMID:26653568, PMID:26721510, PMID:26760117, PMID:26968342, PMID:27453576, PMID:27459541, PMID:27618111, PMID:27667364, PMID:27720452, PMID:27735078, PMID:27798140,

PMID:27805901, PMID:27810937, PMID:27874831, PMID:27993630, PMID:27995898, PMID:27996939, PMID:28069058, PMID:28069924, PMID:28093478, PMID:28111200, PMID:28132841, PMID:28190767, PMID:28197549, PMID:28219480, PMID:28296634, PMID:28323958, PMID:28324005, PMID:28325473, PMID:28342823, PMID:28343983, PMID:28347402, PMID:28350986, PMID:28376279, PMID:28377582, PMID:28399412, PMID:28414095, PMID:28432138, PMID:28452069, PMID:28467300, PMID:28467933, PMID:28472309, PMID:28475894, PMID:28479060, PMID:28479141, PMID:28489286, PMID:28500754, PMID:28552558, PMID:28575672, PMID:28609657, PMID:28622511, PMID:28630256, PMID:28666573, PMID:28725178, PMID:28733667, PMID:28735753, PMID:28757203, PMID:28757207, PMID:28768177, PMID:28796285, PMID:28843484, PMID:28875930, PMID:28898697, PMID:28899916, PMID:28911166, PMID:28918935, PMID:28920576, PMID:28921568, PMID:28938426, PMID:28977598, PMID:28982707, PMID:29045901, PMID:29056294, PMID:29103956, PMID:29107534, PMID:29107990, PMID:29126185, PMID:29143738, PMID:29161595, PMID:29180260, PMID:29195073, PMID:29195811, PMID:29196315, PMID:29202928, PMID:29211853, PMID:29222907, PMID:29227245, PMID:29246444, PMID:29251594, PMID:29282717, PMID:29290465, PMID:29313490, PMID:29326556, PMID:29396040, PMID:29397366, PMID:29398651, PMID:29429900, PMID:29429926, PMID:29452855, PMID:29458059, PMID:29502955, PMID:29503074, PMID:29504938, PMID:29533771, PMID:29557783, PMID:29570780, PMID:29577359, PMID:29606582, PMID:29622465, PMID:29650696, PMID:29689199, PMID:29697373, PMID:29706577, PMID:29759113, PMID:29768163, PMID:29850793, PMID:29861287, PMID:29873699, PMID:29936182, PMID:29963618, PMID:29997615, PMID:30020076, PMID:30033366, PMID:30043751, PMID:30044993, PMID:30052880, PMID:30057310, PMID:30075231, PMID:30078552, PMID:30099050, PMID:30100185, PMID:30100187, PMID:30100261, PMID:30100266, PMID:30107174, PMID:30107177, PMID:30122557, PMID:30122655, PMID:30126838, PMID:30174305, PMID:30181139, PMID:30184507, PMID:30190412, PMID:30205047, PMID:30225356, PMID:30241606, PMID:30244971, PMID:30244973, PMID:30257209, PMID:30264903, PMID:30266038, PMID:30277501, PMID:30281024, PMID:30293774, PMID:30293785, PMID:30304674, PMID:30311190, PMID:30318338, PMID:30332638, PMID:30332639, PMID:30332642, PMID:30355490, PMID:30380148, PMID:30388410, PMID:30393074, PMID:30415923, PMID:30423298, PMID:30428349, PMID:30445039, PMID:30447021, PMID:30447248, PMID:30472188, PMID:30485811, PMID:30515768, PMID:30517626, PMID:30523786, PMID:30527740, PMID:30527742, PMID:30543777, PMID:30581152, PMID:30589944, PMID:30590043, PMID:30590044, PMID:30590051, PMID:30590435, PMID:30595435, PMID:30595531, PMID:30605676, PMID:30613984, PMID:30625330, PMID:30661930, PMID:30661931, PMID:30661989, PMID:30664241, PMID:30673598, PMID:30685224, PMID:30704910, PMID:30712871, PMID:30773316, PMID:30773367, PMID:30784594, PMID:30784596, PMID:30799057, PMID:30799222, PMID:30801659, PMID:30833507, PMID:30846310, PMID:30853218, PMID:30853931, PMID:30865896, PMID:30872738, PMID:30905607, PMID:30905669, PMID:30917317, PMID:30917320, PMID:30927369, PMID:30930054, PMID:30938680, PMID:30943401, PMID:30943403, PMID:30943406, PMID:30945263, PMID:30952813, PMID:30956060, PMID:30974146, PMID:31015514, PMID:31018126, PMID:31042468, PMID:31046408, PMID:31080799, PMID:31090538, PMID:31107240, PMID:31141684, PMID:31150623, PMID:31155351, PMID:31184711, PMID:31185214, PMID:31189097, PMID:31189110, PMID:31197099, PMID:31217166, PMID:31237956, PMID:31257184, PMID:31262711, PMID:31279658, PMID:31287417, PMID:31302152, PMID:31305240, PMID:31322702, PMID:31325514, PMID:31332781, PMID:31353221, PMID:31375460, PMID:31378590, PMID:31398338, PMID:31439839, PMID:31461654, PMID:31504396, PMID:31519825, PMID:31535395, PMID:31540955, PMID:31543446, PMID:31549732, PMID:31578312, PMID:31586034, PMID:31587290, PMID:31641053, PMID:31665636, PMID:31697235, PMID:31721049, PMID:31722427, PMID:31730859, PMID:31733992, PMID:31746486, PMID:31757673, PMID:31801076, PMID:31816056, PMID:31819276, PMID:31822518, PMID:31851930, PMID:31851935, PMID:31866205, PMID:31868167, PMID:31883968, PMID:31898848, PMID:31917470, PMID:31936236, PMID:31940482, PMID:31940485, PMID:31940492, PMID:32017042, PMID:32023458, PMID:32139584, PMID:32152380, PMID:32255424, PMID:32297857, PMID:32312384, PMID:32346865, PMID:32362314, PMID:32365113, PMID:32424151, PMID:32510328, PMID:32663221, PMID:32686647, PMID:32728066, PMID:32773031, PMID:32840210

GFAP polyclonal rabbit, Santa Cruz Biotechnology; (RRID: AB_627673)
Target:
GFAP (2E1) bovine, mouse, mouse, rat, human, cow, human, rat
Validation:
Validated acoording to the manufacturers homepage.
Publications:
PMID:26271210, PMID:27316329, PMID:27924451, PMID:30168601, PMID:30380151, PMID:30779139, PMID:30861520, PMID:31146332

pERK1/2 polyclonal rabbit cell signaling technology (RRID: AB_331646)
Target:
Erk1/2 chicken, fish, hamster, human, mouse, other, raslightly modified β-cytoplasmic actin N-terminal peptide, Ac-Asp-Asp-Asp-Ile-Ala-Ala-Leu-Val-Ile-Asp-Asn-Gly-Ser-Gly-Lys, conjugated to KLH pig, hirudo medicinalis, bovine, rat, canine, feline, human, rabbit, carp, mouse, guinea pig, chicken, sheept, human, mouse, rat, chicken, hamster, zebrafish
Validation:
According to the manufacturers homepage phospho-p44/42 MAPK (Erk1/2) (Thr202/Tyr204) Antibody detects endogenous levels of p44 and p42 MAP Kinase (Erk1 and Erk2) when phosphorylated either individually or dually at Thr202 and Tyr204 of Erk1 (Thr185 and Tyr187 of Erk2). The antibody does not cross-react with the corresponding phosphorylated residues of either JNK/SAPK or p38 MAP Kinase, and does not cross-react with non-phosphorylated Erk1/2.
Publications:
PMID:16871540, PMID:18196540, PMID:18459137, PMID:19937707, PMID:23959936, PMID:25057190, PMID:25164669, PMID:25164676, PMID:25209287, PMID:25392500, PMID:25639954, PMID:25815422, PMID:25830704, PMID:25885794, PMID:26125466, PMID:26132918, PMID:26153447, PMID:26200092, PMID:26284425, PMID:26327577, PMID:26601776, PMID:26919384, PMID:26990063, PMID:27035653, PMID:27253999, PMID:27475840, PMID:27546836, PMID:27569656, PMID:27574484, PMID:27644154, PMID:27765467, PMID:27845624, PMID:27863244, PMID:27881782, PMID:27886513, PMID:28005008, PMID:28009527, PMID:28077724, PMID:28094001, PMID:28100749, PMID:28132840, PMID:28212752, PMID:28235199, PMID:28244869, PMID:28324027, PMID:28332979, PMID:28340336, PMID:28349660, PMID:28374012, PMID:28380381, PMID:28388415, PMID:28399411, PMID:28414095, PMID:28451639, PMID:28463110, PMID:28479141,

PMID:28547849, PMID:28697340, PMID:28736134, PMID:28752899, PMID:28787589, PMID:28801231, PMID:28832322, PMID:28843779, PMID:28877457, PMID:28890319, PMID:28921694, PMID:28977593, PMID:28988823, PMID:29053960, PMID:29056512, PMID:29056551, PMID:29058672, PMID:29097596, PMID:29112861, PMID:29126263, PMID:29134561, PMID:29153842, PMID:29155981, PMID:29173281, PMID:29225023, PMID:29232556, PMID:29245011, PMID:29248508, PMID:29249692, PMID:29249695, PMID:29257952, PMID:29264472, PMID:29275861, PMID:29291977, PMID:29337666, PMID:29358084, PMID:29366902, PMID:29390112, PMID:29395869, PMID:29396039, PMID:29396160, PMID:29410497, PMID:29444261, PMID:29483280, PMID:29510700, PMID:29547721, PMID:29550255, PMID:29635291, PMID:29649442, PMID:29663362, PMID:29677490, PMID:29706577, PMID:29720552, PMID:29739364, PMID:29747239, PMID:29804890, PMID:29856313, PMID:29980436, PMID:29987482, PMID:29989546, PMID:30009811, PMID:30017354, PMID:30049452, PMID:30054205, PMID:30057274, PMID:30076211, PMID:30078576, PMID:30082067, PMID:30126838, PMID:30130530, PMID:30146158, PMID:30174307, PMID:30205046, PMID:30231985, PMID:30244971, PMID:30248305, PMID:30269950, PMID:30269989, PMID:30300582, PMID:30318355, PMID:30320919, PMID:30332648, PMID:30347438, PMID:30373770, PMID:30404005, PMID:30406188, PMID:30449325, PMID:30454647, PMID:30458137, PMID:30517860, PMID:30535142, PMID:30595532, PMID:30607903, PMID:30612939, PMID:30686755, PMID:30700530, PMID:30712867, PMID:30773367, PMID:30801659, PMID:30840890, PMID:30840894, PMID:30849374, PMID:30853557, PMID:30858928, PMID:30880156, PMID:30885633, PMID:30893593, PMID:30893613, PMID:30898653, PMID:30938680, PMID:30956133, PMID:30975481, PMID:31002796, PMID:31031016, PMID:31032919, PMID:31063128, PMID:31078527, PMID:31085102, PMID:31085175, PMID:31141684, PMID:31155483, PMID:31199479, PMID:31217166, PMID:31237611, PMID:31245856, PMID:31291191, PMID:31296909, PMID:31344270, PMID:31353321, PMID:31390566, PMID:31420117, PMID:31433979, PMID:31433983, PMID:31461652, PMID:31484069, PMID:31492153, PMID:31515514, PMID:31541001, PMID:31541095, PMID:31573508, PMID:31586591, PMID:31597089, PMID:31597090, PMID:31597095, PMID:31602645, PMID:31679931, PMID:31730855, PMID:31733992, PMID:31742704, PMID:31746735, PMID:31759919, PMID:31794718, PMID:31894239, PMID:31940493, PMID:31950499, PMID:31968254, PMID:31983610, PMID:32054879, PMID:32096759, PMID:32170720, PMID:32294439, PMID:32342021, PMID:32452765, PMID:32513385, PMID:32513387, PMID:32667883, PMID:32831175, PMID:32873857

Synapsin rabbit polyclonal, Millipore, (RRID:AB_2200400)
Target:
Synapsin I human, mouse, rat, bovine
Validation:
According to the manufacturers homepage Immunolabeling is blocked by preadsorption of antibody with Synapsin I. Synapsin is located only in brain nerve terminal and is an excellent marker for synapses.
Publications:
PMID:21800318, PMID:23749657, PMID:23787772, PMID:24966368, PMID:25560461, PMID:27521497, PMID:27986924, PMID:28041852, PMID:28093476, PMID:28546101, PMID:28955722, PMID:29024660, PMID:29200999, PMID:29540552, PMID:29798891, PMID:29981480, PMID:30125942, PMID:30220511, PMID:30308166, PMID:30578767, PMID:30861124, PMID:31471472, PMID:31711782, PMID:32415988

pSTAT3 monoclonal, rabbit, (RRID:AB_2491009)
Target:
Phospho-Stat3 (Tyr705): mouse, non-human primate, rat, bovine, porcine, hamster, human, mice c57bl/6xcba/caj hybrid, h, m, r, mk, (hm, b, pg, hr), horse
Validation:
Validated in western blot and immunohistochemistry according to the manufacturer's website:
Applications: W, IP, IHC-P, IHC-F, IF-IC, F, ChIP, ChIP-seq. Consolidation on 9/2016: AB_561305, AB_561307, AB_10140371. D3A7 rabbit Cell Signaling Technology 9145 also 9145S, 9145P, 9145L
Moreover, staining with the pSTAT3 antibody showed typical nuclear localization and was validated in our previous studies, for example, by the absence of the staining in conditional STAT3-/- mice after inflammatory stimulation (Leibinger et al., 2013)
Publications
PMID:18615534, PMID:20017211, PMID:23696567, PMID:23698719, PMID:23751875, PMID:24428531, PMID:24742194, PMID:25057790, PMID:25521581, PMID:25814555, PMID:26168341, PMID:26420542, PMID:26671183, PMID:27183315, PMID:27679811, PMID:27939431, PMID:28288167, PMID:28323920, PMID:28399411, PMID:28434617, PMID:28768176, PMID:28771710, PMID:28771727, PMID:28919040, PMID:28938472, PMID:29029019, PMID:29113698, PMID:29148109, PMID:29153838, PMID:29198941, PMID:29222114, PMID:29249690, PMID:29275959, PMID:29358084, PMID:29398601, PMID:29398651, PMID:29458059, PMID:29511338, PMID:29522093, PMID:29533785, PMID:29562202, PMID:29653697, PMID:29659786, PMID:29804891, PMID:29805100, PMID:29887318, PMID:29909986, PMID:29910149, PMID:29993362, PMID:30029001, PMID:30040142, PMID:30068990, PMID:30100196, PMID:30170813, PMID:30205046, PMID:30216497, PMID:30230471, PMID:30259980, PMID:30282020, PMID:30292429, PMID:30304679, PMID:30332632, PMID:30343896, PMID:30415998, PMID:30446383, PMID:30463015, PMID:30472020, PMID:30541071, PMID:30605687, PMID:30686755, PMID:30713111, PMID:30735121, PMID:30797773, PMID:30840056, PMID:30917307, PMID:30930146, PMID:30970258, PMID:30975481, PMID:30990169, PMID:31030416, PMID:31053533, PMID:31067461, PMID:31091445, PMID:31104942, PMID:31199472, PMID:31276158, PMID:31399282, PMID:31429823, PMID:31494101, PMID:31554967, PMID:31555819, PMID:31597099, PMID:31708446, PMID:31715132, PMID:31739488, PMID:31741772, PMID:31825815, PMID:31875548, PMID:31917470, PMID:31944423, PMID:31955990, PMID:31968254, PMID:31983610, PMID:9305919

STAT3 polyclonal, rabbit, (RRID:AB_2629499)
Target:
Human, Mouse, Rat, Monkey
Validation:
Validated in western blot and immunohistochemistry according to the manufacturer's website:

"Stat3 (D3Z2G) Rabbit mAb recognizes endogenous levels of total Stat3 protein. Species cross-reactivity for IF-IC and F is human only." Applications: W, IP, IHC-P, IHC-F, IF-IC, F, ChIP, ChIP-seq.
Publications:
PMID:28826481, PMID:29113698, PMID:29222114, PMID:29562202, PMID:29894692, PMID:30686755, PMID:30865888, PMID:30917307, PMID:30930146, PMID:31091445, PMID:31104942, PMID:31182472, PMID:31555819, PMID:31708446, PMID:31715132, PMID:31801064

Cd11b, rat monoclonal (RRID:AB_324660)
Target:
Rat anti Mouse CD11b antibody, clone M1/70.15 recognizes the murine CD11b cell surface antigen also known as the alpha M integrin chain or MAC-1, a differentiation antigen expressed by granulocytes, monocytes, NK cells and tissue macrophages.
Cross(serotoninreactivity: rabbit, human
Validation:
Validated in western blot and immunohistochemistry according to the manufacturer's website:
"This product has been reported to work in the following applications. This information is derived from testing within our laboratories, peer-reviewed publications or personal communications from the originators. Manufacturer recommendations: Flow Cytometry; Immunohistochemistry; Immunoprecipitation; Immunohistology - Frozen, Immunohistology - Paraffin, Immunoprecipitation, Flow Cytometry"
"Rat anti Mouse CD11b antibody, clone M1/70.15 has been reported to as being suitable for use on PLP fixed paraffin embedded tissue but has not been tested for use on formalin fixed tissue (Whiteland et al. 1995)."
Publications:
PMID:28270575, PMID:30252950, PMID:30394585, PMID:31479164

IL-6 polyclonal, rabbit (RRID:AB_2127460)
Target:
IL6 human, mouse
Validation:
The antibody showed only a signal in cell bodies and axons of hyper- IL-6 transduced but control mice after cortical viral delivery. This was also validated by overexpression in retinal ganglion cells or HEK2903 cells in our previous publication (Leibinger et al. 2016).
Publications
PMID:24684302, PMID:26214037, PMID:28340339, PMID:28683288, PMID:29398596, PMID:29423951, PMID:30860873, PMID:30943581, PMID:31969555, PMID:32412785

polyclonal HA antibody (RRID:AB_260070):
Validation:
Validated in western blot and immunohistochemistry according to the manufacturer's website:
"Anti-HA antibody is suitable for use in immunoprecipitation and western blot. (Ludwig M, et al. Human Genetics 117(2-3), 228-237, (2005); Iris H Henn et. al The Journal of neuroscience, 27(8), undefined (2007-2-23))
It is also suitable for indirect immunofluorescence (10-20µg/mL using HA-tagged fusion protein transfected cells), immunoprecipitation (2.5-4µg/test using HA-tagged fusion protein from cell lysates), and western blot (0.5-0.8µg/mL using HA-tagged fusion protein transfected cell extracts)."
Target:
HA human
Publications:
PMID:21830221, PMID:27538435, PMID:27637097, PMID:27725085, PMID:28630333, PMID:28841137, PMID:28951451, PMID:29024645, PMID:29024665, PMID:29073373, PMID:29154130, PMID:29290488, PMID:29290611, PMID:29649442, PMID:29677490, PMID:29681460, PMID:29934346, PMID:29983322, PMID:30044983, PMID:30078703, PMID:30231999, PMID:30244971, PMID:30281024, PMID:30293784, PMID:30404010, PMID:30404013, PMID:30415698, PMID:30550789, PMID:30612859, PMID:30784600, PMID:30995488

polyclonal GFP antibody (RRID:AB_10128178):
Validation:
Seller suggested use: ELISA; Immunohistochemistry; Immunohistochemistry - fixed; Immunofluorescence; Western Blot; ELISA, Immunofluorescence, Immunohistochemistry, Immunohistochemistry-Paraffin, Western Blot.
Publications:
PMID:28600222, PMID:28630333, PMID:28641114, PMID:29222469, PMID:29587142, PMID:30454645, PMID:30982771

monoclonal pS6 antibody (RRID:AB_2181035):
Validation:
Validated according to manufacturer's homepage by "western blot analysis of extracts from NIH/3T3 cells, untreated or PDGF-treated (100ng/ml, 20 min)".
Seller suggested use: Western Blot, Immunofluorescence, Immunohistochemistry, Immunohistochemistry-Paraffin.
Target:
Phospho-S6 Ribosomal Protein (Ser235/236) See NCBI gene human, mouse, rat
Publications:
PMID:25100599, PMID:27641504, PMID:28641110, PMID:28817801, PMID:28880149, PMID:29695414, PMID:29727620,

PMID:30673598

monoclonal βIII-tubulin antibody (RRID:AB_2313773):
Validation:
According to manufacturer's homepage "This antibody is well characterized and highly reactive to neuron specific Class III β-tubulin (βIII). TUJ1 does not identify β-tubulin found in glial cells. TUJ1 recognizes an epitope located within the last 15 C-terminal residues.
Nishimura K, et al. 2017. PLoS One. 12(1): e0170568. (ICC)
Jongbloets J, et al. 2017. Nat Commun. 8: 14666. (ICC) PubMed
Liu W.J, et al. 2015. Eur J Histochem. 59(1): 2464. (ICC) PubMed
Chintalapudi SR, et al. 2016. Front Aging Neursci. 8:93. (FC, ICC) PubMed
Ambasudhan R, et al. 2011. Cell Stem Cell. 9(2):113. (IHC, ICC)
Hu X., et al. 2006. Nature Neuroscicene. 9(12):1520. (WB) PubMed
Zechner D., et al. 2003. Develop Biology. 258(2):406. (ICC, IHC)
Lee MK, et al. 1990. Proc. Natl. Acad. Sci. USA 18:7195. (WB)"
Target:
Neuronal Class III beta-Tubulin (TUJ1) Purified mammalian, other mammalian, hamster, sheep, bovine, horse, rabbit, guinea pig, human, non-human primate, donkey, feline, goat, porcine, canine, mouse, rat
Publications:
PMID:16680766, PMID:16786555, PMID:16977618, PMID:17154269, PMID:17335037, PMID:17436285, PMID:18205207, PMID:18271024, PMID:18551532, PMID:18651636, PMID:18729150, PMID:18803239, PMID:19350672, PMID:19399895, PMID:19479999, PMID:20017208, PMID:20506477, PMID:21344404, PMID:21452215, PMID:22522921, PMID:22806400, PMID:22847514, PMID:23640803, PMID:25795781, PMID:25961839, PMID:26898779, PMID:27018986, PMID:27418162, PMID:27644593, PMID:27779093, PMID:28009275, PMID:28079521, PMID:28111074, PMID:28123024, PMID:28132826, PMID:28238547, PMID:28340341, PMID:28377582, PMID:28380383, PMID:28426964, PMID:28440222, PMID:28457792, PMID:28483977, PMID:28512649, PMID:28535372, PMID:28539419, PMID:28543060, PMID:28552557, PMID:28630333, PMID:28641113, PMID:28669631, PMID:28789474, PMID:28817799, PMID:28943241, PMID:28965825, PMID:29024661, PMID:29034884, PMID:29151587, PMID:29168882, PMID:29207259, PMID:29218724, PMID:29225067, PMID:29249360, PMID:29249622, PMID:29290548, PMID:29503187, PMID:29551301, PMID:29631040, PMID:29656178, PMID:29660608, PMID:29677589, PMID:29684900, PMID:29706593, PMID:29712777, PMID:29749639, PMID:29784083, PMID:29788427, PMID:29807259, PMID:29853629, PMID:29854941, PMID:29887339, PMID:29906669, PMID:29911975, PMID:29934351, PMID:29961574, PMID:29974865, PMID:30017396, PMID:30057116, PMID:30078710, PMID:30086304, PMID:30099334, PMID:30134160, PMID:30184491, PMID:30225353, PMID:30232223, PMID:30246867, PMID:30252950, PMID:30308165, PMID:30318302, PMID:30343101, PMID:30344048, PMID:30415925, PMID:30449657, PMID:30454561, PMID:30483071, PMID:30485816, PMID:30503143, PMID:30562514, PMID:30562574, PMID:30625321, PMID:30644360, PMID:30650353, PMID:30661738, PMID:30673598, PMID:30697732, PMID:30699345, PMID:30699346, PMID:30735633, PMID:30739799, PMID:30770246, PMID:30846309, PMID:30878013, PMID:30878014, PMID:30891830, PMID:30893594, PMID:30905607, PMID:30921587, PMID:30943410, PMID:31030416, PMID:31042147, PMID:31067457, PMID:31091456, PMID:31099332, PMID:31124784

5-HT (serotonin) (RRID:AB_572262)
Target:
Antigen: Serotonin coupled to BSA with paraformaldehyde. Reacts with:
Cat, Cow, Crayfish, Dolphin, Fish, Frog, Guinea Pig, Hamster, Human, Mexican salamander, Monkey, Moth, Mouse, Pig, Rabbit, Ram, Rat, Salamander, Snail, Tapeworm, Worm, Zebrafish
Validation:
According to manufacturer's homepage, the ImmunoStar Serotonin antiserum was quality control tested using standard immunohistochemical methods. The antiserum demonstrates significant labeling of rat hypothalamus and spinal cord using indirect immunofluorescent and biotin/avidin-HRP techniques. Staining is completely eliminated by pretreatment of the diluted antibody with 100 µg of serotonin/BSA conjugate per mL of diluted antiserum.
Publications:
PMID:10739024, PMID:12031345, PMID:12047717, PMID:12486181, PMID:12581166, PMID:12670312, PMID:1306687, PMID:1375207, PMID:14597586, PMID:14688217, PMID:14705146, PMID:14986100, PMID:15804505, PMID:16354622, PMID:1696730, PMID:17855620, PMID:18054570, PMID:18511045, PMID:18938163, PMID:18950659, PMID:19321769, PMID:19460635, PMID:19496067, PMID:19968985, PMID:20401750, PMID:20696184, PMID:20731710, PMID:20959132, PMID:20959529, PMID:21113792, PMID:21411645, PMID:21411671, PMID:21635768, PMID:21799685, PMID:22064698, PMID:22821666, PMID:22996658, PMID:23041323, PMID:23123789, PMID:23296877, PMID:23922709, PMID:24107617, PMID:24304333, PMID:24421180, PMID:24954002, PMID:25477786, PMID:25586499, PMID:25813788, PMID:2642006, PMID:26560074, PMID:28035899, PMID:28430903, PMID:28602690, PMID:28719042, PMID:29502968, PMID:31309318, PMID:3311380, PMID:3416948, PMID:7532700, PMID:7890832, PMID:8089185, PMID:8440965, PMID:8682935, PMID:8815927, PMID:8900169, PMID:9024619, PMID:9040221, PMID:9138248, PMID:9151749, PMID:9312129, PMID:9324355, PMID:9357469, PMID:9374708, PMID:9377638, PMID:9477297, PMID:9554992, PMID:9603782, PMID:9751494, PMID:9880043

5-HT (RRID:AB_572263)
Target:
Antigen: Serotonin coupled to BSA with paraformaldehyde. Reacts with:
Amphibian, Ant, Bacteria, Bee, Beetle, Bird, Buffalo, Canine, Chicken, Cockroach, Crab, Dove, Feline, Ferret, Fish, Fly, Frog, Fruit Bat, Gerbil, Gnat, Guinea Pig, Hamster, Hen, Human, Insect, Lizard, Lobster, Moth, Monkey, Mollusca, Mosquito, Mouse, Mudpuppy, Mussel, Newt, Octopus, Pig, Pigeon, Quail, Rabbit, Rat, Scorpion, Slug, Seahorse, Shark, Sheep, Shrew, Snail, Spider, Starfish, Sting Ray, Squid, Tick, Turkey, Turtle, Worm, Zebra Finch, Zebrafish + more

Validation:

According to manufacturer's homepage, the ImmunoStar serotonin antiserum was quality control tested using standard immunohistochemical methods. The antiserum demonstrates strongly positive labeling of rat hypothalamus, raphe nuclei and spinal cord using indirect immunofluorescent and biotin/avidin-HRP techniques. Recommended primary dilution is 1/20,000-1/40,000 in PBS/0.3% Triton X-100 - biotin/avidin-HRP Technique. Staining is completely eliminated by pretreatment of the diluted antibody with 25 ug of serotonin/BSA.

Cross reactivity of Serotonin antisera was examined. With 5µg, 10µg and 25µg amounts the following substances did not react with Serotonin antisera diluted 1/20,000 using the Bn-SA/HRP labeling method: 5-hydroxytryptophan, 5-hydroxyindole -3- acetic acid, and dopamine.

Publications:

PMID:10087030, PMID:10215926, PMID:10341956, PMID:10366650, PMID:10370138, PMID:10393817, PMID:10407057, PMID:10426541, PMID:10498270, PMID:10521780, PMID:10526114, PMID:10575032, PMID:10580859, PMID:10611369, PMID:10614660, PMID:10622438, PMID:10627616, PMID:10632606, PMID:10649828, PMID:10654076, PMID:10662651, PMID:10688875, PMID:10701758, PMID:10713568, PMID:10739024, PMID:10741466, PMID:10754505, PMID:10786822, PMID:10788695, PMID:10867175, PMID:10899929, PMID:10930375, PMID:10973587, PMID:11042596, PMID:11072908, PMID:11135241, PMID:11149386, PMID:11153658, PMID:11158630, PMID:11160435, PMID:11169485, PMID:11173985, PMID:11273930, PMID:11390667, PMID:11406825, PMID:11487658, PMID:11517274, PMID:11544079, PMID:11557597, PMID:11579207, PMID:11675504, PMID:11717367, PMID:11718831, PMID:11739954, PMID:11745628, PMID:11755243, PMID:11782534, PMID:11783913, PMID:11810318, PMID:11818560, PMID:11834589, PMID:11835317, PMID:11842011, PMID:11846004, PMID:11868656, PMID:11896169, PMID:11923026, PMID:11927376, PMID:11954035, PMID:12009624, PMID:12031854, PMID:12106075, PMID:12225691, PMID:12361569, PMID:12401344, PMID:12442313, PMID:12453046, PMID:12454997, PMID:12469125, PMID:12476056, PMID:12480131, PMID:12507391, PMID:12527444, PMID:12561073, PMID:12573710, PMID:12574426, PMID:12596045, PMID:12629679, PMID:12740422, PMID:12756551, PMID:12769454, PMID:12770278, PMID:12809693, PMID:1281170, PMID:12842274, PMID:1285494, PMID:12873389, PMID:12895449, PMID:12966220, PMID:12969029, PMID:1331861, PMID:1349616, PMID:1350068, PMID:1351063, PMID:1361496, PMID:1372173, PMID:1383285, PMID:1413408, PMID:1423521, PMID:1431847, PMID:14514036, PMID:14566945, PMID:14568585, PMID:14596860, PMID:14608665, PMID:14659992, PMID:14667369, PMID:14706534, PMID:14715493, PMID:14725625, PMID:14745106, PMID:14960601, PMID:14991839, PMID:15036624, PMID:15061754, PMID:15126131, PMID:15158078, PMID:15170061, PMID:15184542, PMID:1522253, PMID:15229179, PMID:15240798, PMID:15253805, PMID:15322910, PMID:1541640, PMID:15453992, PMID:15485774, PMID:15504325, PMID:15541873, PMID:15548666, PMID:15574730, PMID:15599613, PMID:15607945, PMID:15626502, PMID:15630449, PMID:15649943, PMID:15654302, PMID:15663475, PMID:15672632, PMID:15689553, PMID:15694921, PMID:15696163, PMID:15758173, PMID:1577119, PMID:15787701, PMID:15803860, PMID:15804402, PMID:1583913, PMID:15858217, PMID:15885216, PMID:15906310, PMID:15925099, PMID:15958737, PMID:15965122, PMID:15972963, PMID:15986410, PMID:16000635, PMID:16051494, PMID:16099035, PMID:16122730, PMID:16123386, PMID:16163845, PMID:16182456, PMID:16193278, PMID:16199035, PMID:16206279, PMID:16219029, PMID:16237167, PMID:16237442, PMID:16263962, PMID:16269527, PMID:16300628, PMID:16341252, PMID:16351821, PMID:16406222, PMID:16414028, PMID:16438966, PMID:16442090, PMID:16469388, PMID:16494908, PMID:16548890, PMID:16549779, PMID:16571630, PMID:16600519, PMID:16603821, PMID:16632206, PMID:1664272, PMID:16689666, PMID:16696855, PMID:16705682, PMID:16728728, PMID:16781815, PMID:16783372, PMID:1679768, PMID:16837581, PMID:16873970, PMID:1690221, PMID:16902991, PMID:16903786, PMID:16917819, PMID:1693764, PMID:16958113, PMID:16959869, PMID:16962678, PMID:16997535, PMID:16998905, PMID:16998939, PMID:17029254, PMID:17032451, PMID:17038668, PMID:17048230, PMID:17059818, PMID:1706343, PMID:1711394, PMID:17125818, PMID:17141961, PMID:17156118, PMID:1716746, PMID:17180703, PMID:17182776, PMID:1719875, PMID:17211625, PMID:1721633, PMID:17222491, PMID:1722490, PMID:1723936, PMID:17258709, PMID:17348014, PMID:17425564, PMID:17428610, PMID:17442747, PMID:17447252, PMID:17508220, PMID:17517679, PMID:17525990, PMID:17526000, PMID:17595538, PMID:17602840, PMID:17611274, PMID:1762682, PMID:17629937, PMID:17650110, PMID:17719577, PMID:17721644, PMID:17828260, PMID:17854776, PMID:17855373, PMID:1787183, PMID:17879281, PMID:17879320, PMID:17928523, PMID:17936268, PMID:17947355, PMID:17948307, PMID:17973120, PMID:18040871, PMID:18056009, PMID:18056162, PMID:18174210, PMID:18181152, PMID:18196597, PMID:18215228, PMID:18215231, PMID:18222609, PMID:18272679, PMID:18272695, PMID:18298440, PMID:18329761, PMID:18330924, PMID:18365004, PMID:18388307, PMID:18405885, PMID:18462055, PMID:18511198, PMID:18561908, PMID:18562602, PMID:18565153, PMID:18590553, PMID:18621145, PMID:18647603, PMID:18652386, PMID:18666203, PMID:18683241, PMID:18702689, PMID:18722360, PMID:18799689, PMID:18802724, PMID:18831528, PMID:18837039, PMID:18925650, PMID:18972553, PMID:18987169, PMID:19025991, PMID:19036986, PMID:19038604, PMID:19039008, PMID:19055726, PMID:1906540, PMID:19070605, PMID:19094089, PMID:19096132, PMID:19125588, PMID:1918446, PMID:19184976, PMID:19199291, PMID:19226511, PMID:19253400, PMID:19263059, PMID:19263418, PMID:19304764, PMID:19374548, PMID:19407216, PMID:19418545, PMID:19459220, PMID:19506907, PMID:19515923, PMID:19522780, PMID:19549531, PMID:19552725, PMID:19659409, PMID:1967616, PMID:19679168, PMID:19679722, PMID:19723566, PMID:19728996, PMID:19735478, PMID:1977101, PMID:1981693, PMID:1981998, PMID:19877280, PMID:19884315, PMID:19908250, PMID:19940189, PMID:19968991, PMID:20083105, PMID:20121357, PMID:20130176, PMID:20161763, PMID:20382225, PMID:20433903, PMID:20437523, PMID:20445063, PMID:20484625, PMID:20487953, PMID:20534525, PMID:2054174, PMID:20576032, PMID:20599897, PMID:20604802, PMID:2062572, PMID:20702718, PMID:20809785, PMID:20853514, PMID:20888395, PMID:20976473, PMID:21113724, PMID:21126319, PMID:21126367, PMID:21225801, PMID:21273439, PMID:2134379, PMID:21385956, PMID:21396946, PMID:21400247, PMID:21410871, PMID:2141749, PMID:21443453, PMID:21568687, PMID:21598293, PMID:21600983, PMID:21647706, PMID:21674495, PMID:2172329, PMID:21753849, PMID:21783457, PMID:21858052, PMID:21893064, PMID:21893082, PMID:21893190, PMID:21976490, PMID:22079829, PMID:22214654, PMID:22243800, PMID:22265660, PMID:22285921, PMID:22310098, PMID:22387004, PMID:22445574, PMID:22461086, PMID:22522777, PMID:22532472, PMID:22570716, PMID:22592945, PMID:22609381, PMID:22674259, PMID:22674904, PMID:22699902, PMID:22702392, PMID:22733991, PMID:22740505, PMID:22771417, PMID:22776498, PMID:22844431, PMID:22886450, PMID:22928026, PMID:22934782, PMID:22960115, PMID:22993445, PMID:23095091, PMID:23155053, PMID:23224769, PMID:23238970, PMID:23295060, PMID:23355812, PMID:23376106, PMID:23407865, PMID:23447551, PMID:23466042, PMID:2361651, PMID:2370323, PMID:23748963, PMID:2381521, PMID:23818330, PMID:23861145, PMID:24015275, PMID:24073217, PMID:24086637, PMID:24161751, PMID:24167708, PMID:24178680, PMID:2417931,

PMID:2419794, PMID:2421209, PMID:24260524, PMID:2426333, PMID:2427481, PMID:24281111, PMID:24325300, PMID:24374795, PMID:24452691, PMID:24476105, PMID:2448003, PMID:2458049, PMID:24614598, PMID:24625099, PMID:24630093, PMID:24648204, PMID:2465102, PMID:2467919, PMID:2468399, PMID:2468516, PMID:24695701, PMID:2475217, PMID:2478594, PMID:2499093, PMID:2499111, PMID:25044160, PMID:25234191, PMID:25557150, PMID:2570467, PMID:2573020, PMID:2581171, PMID:25904999, PMID:25921857, PMID:25986676, PMID:26042202, PMID:26156705, PMID:26178754, PMID:26184391, PMID:26225120, PMID:26244086, PMID:26395878, PMID:26426529, PMID:26517971, PMID:26566032, PMID:26630286, PMID:26631478, PMID:27018863, PMID:27491021, PMID:27499084, PMID:27864931, PMID:28074466, PMID:28322190, PMID:28340505, PMID:28508746, PMID:2858422, PMID:2860081, PMID:28649777, PMID:2868381, PMID:2869065, PMID:28719042, PMID:28771712, PMID:2880936, PMID:2880940, PMID:28817801, PMID:2883596, PMID:28867482, PMID:28968659, PMID:28984573, PMID:29075178, PMID:29103613, PMID:2918092, PMID:2918103, PMID:29206101, PMID:29377111, PMID:29379878, PMID:29681516, PMID:2985748, PMID:29891731, PMID:30007046, PMID:30146164, PMID:30281776, PMID:3039416, PMID:30456293, PMID:30574074, PMID:30689201, PMID:30709851, PMID:30790333, PMID:30861118, PMID:31031000, PMID:31199515, PMID:31309318, PMID:3131390, PMID:31376285, PMID:31383255, PMID:31392919, PMID:31427403, PMID:31445030, PMID:3147123, PMID:31577916, PMID:31621907, PMID:31625610, PMID:31784592, PMID:32124731, PMID:3238176, PMID:3282614, PMID:3301919, PMID:3301921, PMID:3311291, PMID:3359901, PMID:3399053, PMID:3497137, PMID:3546623, PMID:3567612, PMID:3668614, PMID:3670593, PMID:3696488, PMID:3736856, PMID:3892374, PMID:3973090, PMID:3995555, PMID:6131259, PMID:6181120, PMID:6191258, PMID:6198030, PMID:6201597, PMID:6206212, PMID:6209246, PMID:6307044, PMID:6349816, PMID:6349819, PMID:6371583, PMID:6378989, PMID:6380643, PMID:6384280, PMID:6384830, PMID:6388735, PMID:6480896, PMID:7049319, PMID:7477901, PMID:7505834, PMID:7511035, PMID:7541167, PMID:7541286, PMID:7570024, PMID:7589323, PMID:7606468, PMID:7609624, PMID:7663967, PMID:7686567, PMID:7688882, PMID:7690669, PMID:7718493, PMID:7739266, PMID:7751436, PMID:7756620, PMID:7768178, PMID:7814671, PMID:7845592, PMID:7845627, PMID:7887054, PMID:7902864, PMID:7908956, PMID:7909312, PMID:7915726, PMID:7931567, PMID:7965063, PMID:7987666, PMID:7996458, PMID:8055350, PMID:8104818, PMID:8113788, PMID:8118841, PMID:8132859, PMID:8182530, PMID:8227147, PMID:8252414, PMID:8264992, PMID:8320484, PMID:8354879, PMID:8364956, PMID:8377933, PMID:8383927, PMID:8432364, PMID:8448674, PMID:8450176, PMID:8462655, PMID:8479605, PMID:8507003, PMID:8527376, PMID:8575634, PMID:8589445, PMID:8613731, PMID:8620837, PMID:8624584, PMID:8655420, PMID:8666346, PMID:8700866, PMID:8876467, PMID:8939052, PMID:8995212, PMID:9001724, PMID:9013424, PMID:9100257, PMID:9154524, PMID:9169544, PMID:9181435, PMID:9183687, PMID:9212446, PMID:9215698, PMID:9240327, PMID:9249585, PMID:9255253, PMID:9295317, PMID:9315907, PMID:9315915, PMID:9320037, PMID:9335425, PMID:9359895, PMID:9362295, PMID:9368767, PMID:9425019, PMID:9427250, PMID:9427312, PMID:9446834, PMID:9473622, PMID:9483543, PMID:9614245, PMID:9619500, PMID:9630509, PMID:9675425, PMID:9763486, PMID:9765360, PMID:9774794, PMID:9786212, PMID:9826131, PMID:9831252, PMID:9832210, PMID:9844013, PMID:9845022, PMID:9870749Publications:

T308-phosphorylated AKT (RRID:AB_2629447):

Validation:

According to manufacturer's homepage, this antibody was validated in "Western blot analysis of extracts from NIH/3T3 cells, untreated (-) or treated with Human Platelet-Derived Growth Factor AA (hPDGF-AA) #8913 (100 ng/ml, 5 min; +), and untreated (-) LNCaP and PC-3 cells. Phospho-Akt (Thr308) (D25E6) XP® Rabbit mAb recognizes endogenous levels of Akt1 protein only when phosphorylated at Thr308. This antibody also recognizes endogenous levels of Akt2 protein when phosphorylated at Thr309 or Akt3 protein when phosphorylated at Thr305. "

Target:

Phospho-Akt (Thr308) Human, Mouse, Rat, Monkey

Publications:

PMID:27792406, PMID:28406396, PMID:28630333, PMID:28757207, PMID:28919041, PMID:29029116, PMID:29246441, PMID:30029001, PMID:30078705, PMID:30126838, PMID:30174303, PMID:30300582, PMID:30566860, PMID:30581121, PMID:30639242, PMID:30704899, PMID:30734931, PMID:30738829, PMID:30773462, PMID:30784600, PMID:31091439

neuN (RRID:AB_2532109)

Target:

Antigen: Synthetic peptide within Human NeuN aa 1-100 (Cysteine residue). The exact sequence is proprietary. Reacts with: Mouse, Rat, Sheep, Goat, Cat, Dog, Human, Pig, Zebrafish, Cynomolgus monkey, Common marmoset

Validation:

Validated according to manufacturer's homepage.

Publications:

PMID:27316329, PMID:27499083, PMID:28632969, PMID:28639376, PMID:28689640, PMID:28965758, PMID:29107533, PMID:29124761, PMID:29279310, PMID:29429934, PMID:29752725, PMID:29954850, PMID:30078577, PMID:30092214, PMID:30125937, PMID:30144320, PMID:30291632, PMID:30318302, PMID:30320555, PMID:30362615, PMID:30378170, PMID:30380151, PMID:30414323, PMID:30421453, PMID:30612898, PMID:30809795, PMID:30827729, PMID:30862665, PMID:30865888, PMID:31267031, PMID:31427401, PMID:31512255, PMID:31628176, PMID:31755578, PMID:31775051, PMID:31785351, PMID:31806492, PMID:31872424, PMID:31951012

Animals and other organisms

Policy information about studies involving animals; ARRIVE guidelines recommended for reporting animal research

Laboratory animals	Male and female mice (2-3 months old) were used. Genotypes: wt mice with C57BL/6,129/Ola, or C57BL/6 background and PTENf/f mice (C57BL/6,129/Ola), or ROSA dtTomao reporter mice
Wild animals	not used
Field-collected samples	not used
Ethics oversight	All experimental procedures were approved by the local animal care committee (LANUV Recklinghausen) and conducted in compliance with federal and state guidelines for animal experiments in Germany.

Note that full information on the approval of the study protocol must also be provided in the manuscript.
